# The Mediterranean Diet as a Sustainable Dietary Pattern: A State-of-the-Art Narrative Review of Health, Environmental and Socioeconomic Dimensions

**DOI:** 10.3390/nu18121925

**Published:** 2026-06-13

**Authors:** Georgios K. Vasios, Maria Gialeli, Georgios Antasouras, Constantinos Giaginis

**Affiliations:** Department of Food Science and Nutrition, School of Environment, University of the Aegean, 81400 Lemnos, Greece; vasios@aegean.gr (G.K.V.); gialeli.m@aegean.gr (M.G.); g.antasouras@gmail.com (G.A.)

**Keywords:** Mediterranean diet, sustainable diets, environmental impact, non-communicable diseases, ultra-processed foods, public health nutrition

## Abstract

**Background/Objectives**: The increasing burden of non-communicable diseases, together with accelerating environmental degradation, highlights the urgent need for sustainable dietary patterns that promote both human and planetary health. The Mediterranean diet (MedDiet), traditionally followed in countries bordering the Mediterranean basin, has gained recognition as a model of sustainable nutrition due to its well-documented health benefits and relatively low environmental impact. However, its broader role within sustainable food systems requires comprehensive and interdisciplinary evaluation. The aim of this review is to provide a state-of-the-art synthesis of the evidence on the MedDiet as a sustainable dietary pattern, integrating its health, environmental, economic, and socio-cultural dimensions. **Methods**: This state-of-the-art narrative review synthesizes evidence from peer-reviewed literature on the MedDiet and sustainability. Relevant studies were identified through major scientific databases, focusing on publications addressing nutritional, environmental, economic, and socio-cultural dimensions. Both observational and interventional studies, as well as modeling and life cycle assessment analyses, were included. Additional sources from international organizations and policy reports were incorporated to contextualize global trends and challenges. **Results**: High adherence to the MedDiet is consistently associated with a reduced risk of cardiovascular disease, type 2 diabetes, cancer, and all-cause mortality. From an environmental perspective, the MedDiet is associated with lower greenhouse gas emissions, reduced land and water use, and enhanced biodiversity conservation compared with Western dietary patterns. Economically, it may represent a cost-effective dietary model and support local food systems when grounded in traditional practices, although affordability varies across contexts. Socio-culturally, the MedDiet promotes food heritage, culinary skills, and social cohesion. Nevertheless, globalization, urbanization, and the increasing consumption of ultra-processed foods have contributed to declining adherence, posing significant challenges to its sustainability and scalability. Moreover, the sustainability benefits of the MedDiet seem to be context-dependent rather than intrinsic, raising several challenges and limitations for its adoption. **Conclusions**: The MedDiet should be viewed not as a definitive solution to global food-system challenges but as a valuable reference model that illustrates how dietary practices can contribute simultaneously to human health, environmental sustainability, and cultural continuity. Modern sustainable dietary strategies should build upon the strengths of the MedDiet while recognizing its limitations, embracing contextual adaptation, and addressing the structural determinants that shape food choices.

## 1. Introduction

The global food system is increasingly recognized as a critical nexus between human health, environmental sustainability, and socioeconomic development. Over recent decades, profound transformations in food production, distribution, and consumption have contributed to both improved food availability and significant unintended consequences. While global caloric supply has increased, these gains have been accompanied by a sharp rise in diet-related non-communicable diseases (NCDs), including cardiovascular disease, type 2 diabetes, obesity, and certain cancers [1,2,3]. Concurrently, food systems have emerged as major drivers of environmental degradation, contributing substantially to greenhouse gas (GHG) emissions, land-use change, freshwater depletion, and biodiversity loss [4,5,6].

It is estimated that food systems account for approximately one-third of global GHG emissions, with livestock production alone contributing a disproportionate share due to methane emissions, feed production, and land conversion [4,7]. Furthermore, agricultural expansion remains the leading cause of deforestation and habitat destruction worldwide, posing a direct threat to biodiversity and ecosystem stability [8]. These environmental pressures are compounded by a growing global population, projected to reach nearly 10 billion by 2050, and shifting dietary preferences toward resource-intensive foods, particularly in low- and middle-income countries undergoing rapid economic transition [9].

At the same time, dietary patterns have shifted dramatically toward what is often described as the “Western diet”, characterized by high consumption of red and processed meats, refined grains, added sugars, saturated fats, and ultra-processed foods [10]. This dietary transition is closely associated with increased energy intake, poor nutrient density, and adverse health outcomes, contributing to the global burden of NCDs [2,11]. The coexistence of undernutrition, micronutrient deficiencies, and obesity—often referred to as the “triple burden of malnutrition”—highlights the complexity of current global nutrition challenges [12].

In response to these interconnected issues, the concept of sustainable diets has gained increasing attention among researchers, policymakers, and international organizations. According to the Food and Agriculture Organization (FAO), sustainable diets are those with low environmental impacts that contribute to food and nutrition security and healthy lives for present and future generations, while being culturally acceptable, economically fair, and accessible [13]. This multidimensional definition underscores the need for integrated approaches that address health, environment, and social equity simultaneously.

Within this context, the Mediterranean diet (MedDiet) has emerged as one of the most widely cited and extensively studied examples of a sustainable dietary pattern. Originally described through epidemiological observations of Mediterranean populations during the mid-20th century, the MedDiet is characterized by a high consumption of fruits, vegetables, legumes, whole grains, nuts, seeds, and olive oil; moderate consumption of fish, seafood, dairy products, and wine; and low consumption of red and processed meats and highly processed foods [14,15,16,17,18,19,20,21,22,23]. From a nutritional and public health perspective, a substantial body of evidence supports the health-promoting effects of the MedDiet. Compared with low adherence, high adherence to the MedDiet has been associated with approximately 24–30% lower cardiovascular mortality, a 20–25% lower incidence of coronary heart disease, and a 15–25% lower risk of stroke in prospective cohort studies and meta-analyses [21,22,23]. Moreover, high MedDiet adherence has consistently been associated with improved metabolic health, reduced incidence of type 2 diabetes, lower rates of obesity, certain cancers, neurodegenerative diseases, and increased longevity [21,22,23]. Landmark studies such as the PREDIMED trial demonstrated significant reductions in major cardiovascular events among individuals following a Mediterranean dietary pattern supplemented with extra-virgin olive oil or nuts [24]. Consequently, the MedDiet is widely regarded as one of the most evidence-based dietary models for chronic disease prevention and healthy aging.

The growing interest in the MedDiet extends beyond its nutritional composition. In recent decades, international organizations including the FAO, the World Health Organization (WHO), and the EAT–Lancet Commission have increasingly emphasized the need for dietary models that simultaneously promote human health, environmental sustainability, economic resilience, and socio-cultural well-being [4,12,13]. Within this framework, the MedDiet is frequently recognized as a reference model for sustainable diets because it aligns with the multidimensional definition proposed by the FAO, which describes sustainable diets as dietary patterns that are nutritionally adequate, environmentally protective, economically accessible, culturally acceptable, and capable of supporting present and future generations [4,12,13].

**Figure 1:**
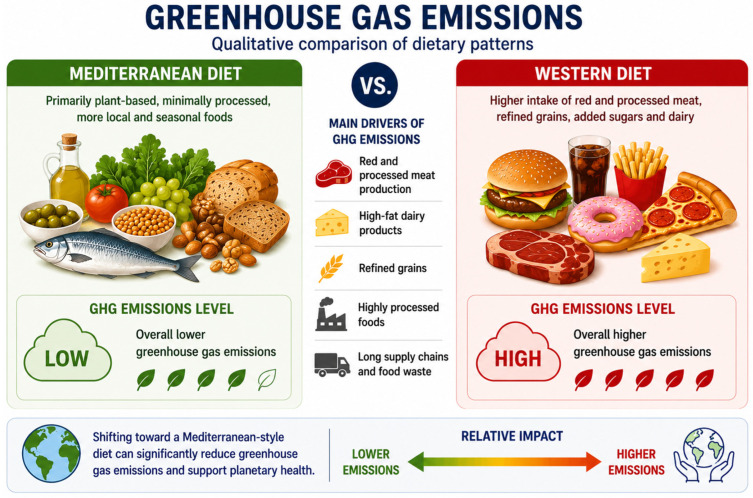
Qualitative comparison of GHG emissions between MedDiet and Western Dieta patterns.

From an environmental perspective, the predominantly plant-based nature of the MedDiet is associated with lower resource requirements and reduced ecological burdens compared with Western dietary patterns characterized by high consumption of animal-source foods. More to the point, higher adherence to the MedDiet has been associated with approximately 20–50% lower GHG emissions compared with typical Western dietary patterns, largely due to reduced consumption of red and processed meats and greater reliance on plant-based foods [25,26,27,28]. In addition, higher adherence to the MedDiet has been linked to lower land and water use, improved resource efficiency, and greater support for biodiversity conservation [25,26,27,28]. The emphasis on seasonal, locally produced foods and shorter supply chains may further contribute to environmental sustainability and food-system resilience. In Figure 1, a qualitative comparison of GHG emissions between MedDiet and Western Dieta patterns is illustrated.

Economic sustainability also represents an important component of the MedDiet framework. Although concerns regarding affordability have been raised in some settings, particularly during periods of economic instability and food inflation, several studies suggest that traditional Mediterranean dietary patterns can be economically sustainable when based on locally available plant foods, legumes, seasonal produce, and minimally processed ingredients [25,26,27,28]. Furthermore, the MedDiet has been associated with broader economic benefits through potential reductions in healthcare expenditures attributable to diet-related chronic diseases and through support of regional agricultural systems and local food economies.

Equally important are the socio-cultural dimensions of the MedDiet. Unlike many dietary recommendations that focus primarily on nutrient intake, the Mediterranean dietary model incorporates cultural heritage, culinary traditions, social interaction, and food-related practices. Shared meals, traditional culinary knowledge, intergenerational transmission of food culture, seasonality, and the preservation of local food systems constitute integral components of the Mediterranean lifestyle [18,19]. These characteristics contributed to the recognition of the MedDiet by UNESCO as an Intangible Cultural Heritage of Humanity and underscore its importance as a socio-cultural model of sustainability [20].

The MedDiet is therefore increasingly viewed not merely as a dietary pattern but as a holistic food-system model that simultaneously addresses the four pillars of sustainable diets: environmental sustainability, nutritional adequacy and health promotion, economic viability, and socio-cultural acceptability. This multidimensional perspective has positioned the MedDiet at the center of contemporary discussions regarding sustainable food-system transformation, planetary health, and climate-resilient nutrition strategies.

Despite these advantages, adherence to the MedDiet has declined substantially over recent decades, even within countries traditionally associated with Mediterranean food cultures. This phenomenon has been attributed to globalization, urbanization, changes in food environments, shifts in lifestyle patterns, increasing consumption of ultra-processed foods, and the progressive adoption of Westernized dietary behaviors [29,30,31]. The erosion of traditional Mediterranean dietary practices raises concerns not only for population health but also for the preservation of cultural heritage, biodiversity, local agricultural systems, and the long-term sustainability of food systems. Consequently, understanding the factors that promote or hinder adherence to the MedDiet has become an increasingly important research and policy priority within the broader framework of sustainable development and planetary health [29,30,31]. Given these considerations, there is a growing need to evaluate the MedDiet within a comprehensive sustainability framework. While numerous studies have examined its individual components, fewer have integrated environmental, nutritional, economic, and socio-cultural dimensions into a unified analysis. Therefore, the aim of this state-of-the-art review is to provide an in-depth and multidimensional examination of the MedDiet as a sustainable dietary pattern. Specifically, this state-of-the-art narrative review aims to: (i) analyze its environmental impact; (ii) evaluate its nutritional and health benefits; (iii) explore its economic and socio-cultural dimensions; and (iv) identify challenges, barriers, limitations and future directions for its adoption in diverse contexts.

## 2. Methods

### 2.1. Review Design

This study was conducted as a state-of-the-art review aimed at critically evaluating the current scientific understanding of the MedDiet as a sustainable dietary pattern and identifying emerging developments, unresolved challenges, and future research priorities. A state-of-the-art review methodology was selected because the topic spans multiple scientific disciplines, including nutrition science, environmental sustainability, public health, economics, food systems, agriculture, and socio-cultural research [32]. The available evidence is highly heterogeneous, encompassing observational studies, randomized controlled trials (RCTs), life-cycle assessment (LCA) studies, economic analyses, policy reports, and qualitative investigations, which limits the applicability of quantitative synthesis methods.

Unlike systematic reviews that seek to answer narrowly defined research questions through exhaustive evidence retrieval, state-of-the-art reviews focus on integrating the most influential, contemporary, and conceptually important evidence to provide an updated perspective on current knowledge, emerging trends, scientific controversies, and future directions [33]. Accordingly, the objective of the present review was to synthesize and critically evaluate current evidence regarding the environmental, nutritional, economic, and socio-cultural dimensions of the MedDiet within the broader context of sustainable food systems.

### 2.2. Literature Search Strategy

A comprehensive literature search was conducted between January and March 2026, with the final search update performed on 31 March 2026. The electronic databases PubMed/MEDLINE, Scopus, and Web of Science were systematically searched to identify relevant literature. Additional sources were identified through manual screening of reference lists from key articles, consensus statements, landmark reviews, and policy documents, as well as reports published by international organizations including the Food and Agriculture Organization of the United Nations (FAO), the World Health Organization (WHO), and the EAT–Lancet Commission [4,34].

#### 2.2.1. Time Frame and Language Restrictions

All searches were limited to peer-reviewed English-language publications published between January 1995 and March 2026. The starting date was selected to capture both the historical development of the MedDiet as a recognized healthy dietary pattern and the emergence of sustainability as a central concept in nutrition, environmental science, and food-system research. Although particular emphasis was placed on contemporary evidence, especially studies published within the last three decades, seminal publications were retained when considered essential for understanding the conceptual foundations, methodological evolution, and policy relevance of the field.

#### 2.2.2. Search Terms and Conceptual Domains

Search terms were developed to capture the multidimensional nature of the MedDiet and its relationship with sustainable food systems. The search strategy combined keywords, controlled vocabulary terms (MeSH where applicable), and Boolean operators across five major thematic domains: (i) dietary patterns, (ii) environmental sustainability, (iii) health outcomes, (iv) economic sustainability, and (v) socio-cultural dimensions. The dietary pattern domain included terms such as: (“Mediterranean diet” OR “Mediterranean dietary pattern” OR “Mediterranean-style diet” OR “traditional Mediterranean diet”). The sustainability domain included: (“sustainable diet*” OR “dietary sustainability” OR “sustainable nutrition” OR “sustainable food system*” OR “food system sustainability” OR “sustainable consumption” OR “sustainable agriculture”). The environmental domain included: (“environmental sustainability” OR “environmental impact” OR “ecological impact” OR “greenhouse gas emission*” OR GHG OR “carbon footprint” OR “water footprint” OR “land use” OR “resource use” OR “energy use” OR “climate change” OR “climate resilience” OR biodiversity OR ecosystem* OR “ecosystem services” OR “environmental footprint”). The health and nutrition domain included: (“public health nutrition” OR “diet quality” OR “nutritional adequacy” OR “healthy eating” OR “chronic disease prevention” OR cardiovascular OR obesity OR diabetes OR cancer OR longevity OR “healthy ageing” OR “population health”). The economic domain included: (“economic sustainability” OR affordability OR accessibility OR “food affordability” OR “food cost*” OR “cost-effectiveness” OR “economic impact” OR “food security” OR “food sovereignty” OR “food availability”). The socio-cultural domain included: (“cultural heritage” OR “food culture” OR “traditional food systems” OR “culinary traditions” OR “food literacy” OR “consumer behavior” OR “dietary transition” OR westernization OR “social sustainability” OR “community resilience”).

#### 2.2.3. Emerging Sustainability Concepts

To capture emerging concepts linking nutrition and sustainability, additional terms were incorporated, including: (“planetary health” OR “planetary health diet” OR “One Health” OR “food-system transformation” OR “sustainable development” OR SDGs OR “United Nations Sustainable Development Goals”).

#### 2.2.4. Additional Representative Boolean Search Combinations

Additional representative Boolean search combinations included: (“Mediterranean diet” OR “Mediterranean dietary pattern”) AND (“sustainable diet*” OR “dietary sustainability” OR “sustainable food system*”) AND (“environmental impact” OR “greenhouse gas emissions” OR “carbon footprint” OR biodiversity OR “food security” OR “planetary health”) and (“Mediterranean diet”) AND (“public health nutrition” OR “diet quality” OR “chronic disease prevention”) AND (“sustainability” OR “sustainable development” OR “food-system transformation”).

The search strategy was intentionally broad to ensure comprehensive coverage of environmental, nutritional, economic, and socio-cultural dimensions of sustainability while also capturing emerging themes related to planetary health, climate resilience, and sustainable food-system transformation.

#### 2.2.5. Database-Specific Adaptations and Supplementary Searches

Additional database-specific search strategies were adapted for Scopus and Web of Science according to their indexing systems and search functionalities. Furthermore, targeted searches were conducted for specific sustainability domains, including environmental sustainability, climate change mitigation, biodiversity conservation, food-system resilience, public health outcomes, economic sustainability, cultural heritage, and policy implementation. Manual searches of reference lists from highly cited reviews, systematic reviews, meta-analyses, and international policy reports were also performed to identify influential studies not captured through electronic database searches.

#### 2.2.6. Temporal Coverage of the Literature

Particular emphasis was placed on literature published during the three decades (1995–2026), reflecting recent scientific advances in sustainable diets, planetary health, and food-system transformation. However, influential earlier publications were incorporated where necessary to provide historical context and support the interpretation of current evidence.

### 2.3. Evidence Selection and Data Extraction

Because this review was conducted as a state-of-the-art review rather than a systematic review, evidence selection was guided primarily by scientific relevance, methodological rigor, influence within the field, recency of publication, and contribution to current understanding of sustainable diets. Eligible sources included: Observational studies; RCTs and intervention studies; Systematic reviews and meta-analyses; Umbrella reviews and consensus statements; LCA studies; Economic analyses; Reports from international organizations (e.g., FAO, WHO, EAT–Lancet); Seminal and highly cited publications. Publications were excluded if they: Were not directly related to the MedDiet or sustainability; Lacked sufficient methodological rigor; Were superseded by more recent and comprehensive evidence; Consisted primarily of opinion-based commentary without supporting evidence. For each study, the following information was extracted: Study design and objectives; Population characteristics and geographical setting; Sustainability dimension(s) investigated; Principal findings and conclusions; Methodological strengths and limitations; Relevance to current scientific and policy discussions. Final study selection was conducted by two reviewers (G.K.V. and C.G.).

**Figure 2:**
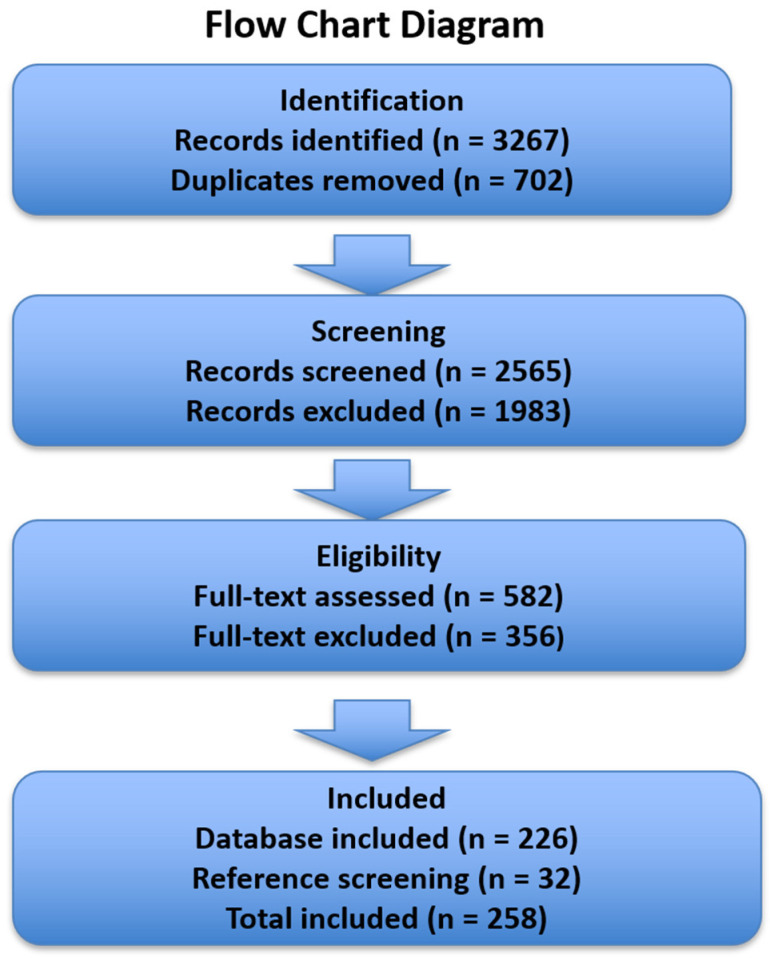
Flow chart diagram of studies enrollment.

The initial search strategy identified 3267 records across all databases. Following removal of duplicate entries (*n* = 702), 2564 records remained eligible for screening. Title and abstract screening resulted in exclusion of non-relevant studies, yielding 582 full-text articles assessed for eligibility. Of these, 226 studies satisfied the predefined inclusion criteria through database screening. An additional 32 studies were identified via manual searching of reference lists, culminating in a final dataset comprising 258 studies included in the qualitative synthesis [32,33]. In Figure 2, the flow chart diagram of studies enrollment is depicted.

### 2.4. Evidence Evaluation and Thematic Synthesis

The selected literature was synthesized qualitatively using a thematic framework based on the multidimensional concept of sustainable diets proposed by the FAO [34]. Evidence was organized into five major thematic domains: (i) Environmental sustainability; (ii) Nutritional and health outcomes; (iii) Economic sustainability; (iv) Socio-cultural dimensions; (v) Barriers to adoption and implementation.

Given the multidisciplinary nature of the topic, evidence was evaluated using a domain-specific approach. Greater interpretative weight was assigned to systematic reviews, meta-analyses, umbrella reviews, and large prospective cohort studies when discussing nutritional and health outcomes. Randomized controlled trials were prioritized when assessing causal relationships between dietary adherence and clinical outcomes.

For environmental sustainability, emphasis was placed on LCA studies, environmental footprint analyses, and comparative food-system evaluations. Economic conclusions were primarily informed by affordability studies, cost-effectiveness analyses, and assessments of food-system resilience. Socio-cultural dimensions were synthesized from population surveys, qualitative studies, ethnographic research, and policy analyses.

Rather than applying formal quality-scoring tools, studies were critically evaluated according to methodological robustness, sample size, analytical rigor, consistency with existing evidence, and relevance to contemporary scientific discourse. When conflicting findings were encountered, greater emphasis was placed on evidence derived from higher-quality study designs and comprehensive evidence syntheses.

In addition to summarizing current knowledge, particular attention was devoted to identifying emerging trends, unresolved controversies, knowledge gaps, and future research directions relevant to sustainable dietary systems.

### 2.5. Methodological Considerations and Limitations

As a state-of-the-art review, this study was not designed as a systematic review and therefore did not involve protocol registration, exhaustive study retrieval procedures, formal risk-of-bias assessment, or quantitative meta-analysis. Consequently, the possibility of selection bias cannot be entirely excluded.

However, several measures were implemented to enhance transparency and scientific rigor, including the use of multiple databases, inclusion of multidisciplinary evidence sources, prioritization of high-quality studies, explicit selection criteria, and critical evaluation of both supporting and contradictory findings. Furthermore, evidence was integrated across environmental, nutritional, economic, and socio-cultural domains to provide a balanced and comprehensive overview of the current state of knowledge regarding the MedDiet as a sustainable dietary pattern.

These methodological choices are consistent with the objectives of state-of-the-art reviews, which aim to provide a critical, forward-looking synthesis of the most influential and contemporary evidence while identifying emerging challenges and future priorities for research and policy development.

## 3. The Mediterranean Diet: Characteristics, Evolution, and Conceptual Foundations

The MedDiet represents more than a set of dietary guidelines; it is a historically grounded and culturally embedded way of life shaped by centuries of interaction between people, food systems, and the Mediterranean environment. This section explores the origins, evolution, and defining characteristics of the MedDiet, tracing its development from ancient civilizations to its modern scientific recognition. It further examines the core dietary components and lifestyle practices that underpin its health benefits, emphasizing the central role of plant-based foods, traditional culinary methods, and social and cultural dimensions. Together, these elements illustrate the MedDiet as a holistic and adaptable model that integrates nutrition, culture, and sustainability.

### 3.1. Historical Origins

The MedDiet is widely recognized not as a static dietary prescription but as a dynamic and evolving dietary pattern, shaped over millennia by the interaction between human populations, agricultural practices, and ecological constraints within the Mediterranean basin [35,36,37]. This region, characterized by its unique climate—mild, wet winters and hot, dry summers—has historically supported the cultivation of drought-resistant crops such as olives, grapes, legumes, and cereals, which form the nutritional backbone of the MedDiet [38,39,40,41].

The historical roots of the MedDiet can be traced to ancient civilizations, including the Greek, Roman, and Arab cultures, which contributed to the diffusion of agricultural techniques, culinary traditions, and food preservation methods across the region [38,39,40,41]. Trade routes throughout the Mediterranean region facilitated the exchange of ingredients and knowledge, reinforcing dietary patterns based on plant-derived foods and modest consumption of animal products. Central to this dietary system is the so-called “Mediterranean triad”—olive oil, wheat, and wine—which has persisted as a defining feature of regional food culture and continues to underpin modern interpretations of the diet [42,43].

Scientific recognition of the MedDiet emerged in the mid-20th century through the pioneering work of Ancel Keys and colleagues in the Seven Countries Study, which remains one of the most influential epidemiological investigations in nutrition science [44,45]. This study demonstrated that populations in Mediterranean regions—particularly in Crete and southern Italy—exhibited significantly lower rates of cardiovascular disease (CVD) and all-cause mortality compared to populations in Northern Europe and the United States, despite relatively high total fat intake. Importantly, this paradox was attributed to the quality rather than quantity of dietary fat, with a predominance of monounsaturated fatty acids derived from olive oil [15,46,47].

Beyond dietary composition, the populations studied also exhibited distinct lifestyle characteristics, including high levels of occupational and recreational physical activity, strong family and community ties, and limited exposure to processed foods [15,46,47,48]. These lifestyle factors collectively contributed to a holistic health-promoting environment, characterized by regular physical movement embedded in daily life, shared meals that reinforce social cohesion, and dietary habits centered on minimally processed, locally sourced foods. The synergistic interaction between diet and lifestyle behaviors is therefore considered a key determinant of the protective effects observed, underscoring that the MedDiet should be understood not merely as a set of foods, but as part of a broader socio-cultural and behavioral framework that supports long-term health outcomes and disease prevention [15,46,47,48].

Over subsequent decades, the MedDiet evolved from an observational construct into a quantifiable and standardized dietary model. The development of adherence indices, such as the MedDiet Score and alternate MedDiet indices, enabled researchers to systematically evaluate associations between diet and health outcomes across diverse populations [21,49,50]. These tools have been instrumental in demonstrating consistent inverse relationships between MedDiet adherence and the risk of chronic diseases, including CVD, type 2 diabetes, and certain cancers [21,49,50].

In recent years, the conceptualization of the MedDiet has expanded further to incorporate principles of sustainability and planetary health. Global initiatives, including the EAT–Lancet Commission, have identified the MedDiet as a reference model for diets that simultaneously promote human health and minimize environmental degradation [4,51,52]. This reframing reflects growing recognition that dietary patterns must be evaluated not only in terms of nutritional adequacy but also in relation to their ecological footprint and long-term viability.

### 3.2. Core Dietary Components

The MedDiet is characterized by a holistic dietary pattern emphasizing whole, minimally processed foods, balanced macronutrient distribution, and a high density of naturally occurring bioactive compounds [24,53,54]. Importantly, its beneficial health effects are not attributable to individual nutrients alone but rather to the synergistic interactions among dietary components, often described as the “food matrix” effect [55,56]. This paradigm underscores the importance of dietary patterns over reductionist nutrient-based approaches in nutrition science.

#### 3.2.1. Plant-Based Foods

Plant-based foods constitute the cornerstone of the MedDiet, accounting for the majority of daily energy intake [57,58]. Epidemiological and clinical evidence consistently demonstrates that high consumption of these foods is associated with reduced risk of chronic diseases and improved overall health outcomes [59,60,61].

Fruits and vegetables provide a wide array of bioactive compounds, including antioxidants such as vitamin C, vitamin E, carotenoids (e.g., β-carotene, lutein, and lycopene), and a diverse range of polyphenols (e.g., flavonoids, phenolic acids, and anthocyanins), as well as dietary fiber, potassium, folate, and other essential micronutrients, which collectively play a crucial role in mitigating oxidative stress and chronic low-grade inflammation—key processes in the pathogenesis of NCDs [59,60,61]. The synergistic interactions among these compounds, including fiber–polyphenol and vitamin–mineral interactions, further enhance their bioavailability and biological activity, reinforcing the importance of whole-food consumption rather than isolated nutrient intake [59,60,61].

Whole grains contribute complex carbohydrates, resistant starch, and fermentable dietary fibers that support gut microbiota diversity and functionality [62,63,64]. These substrates are metabolized by colonic microbiota to produce short-chain fatty acids (SCFAs), including acetate, propionate, and butyrate, which play a central role in maintaining intestinal barrier integrity, regulating immune responses, and modulating systemic metabolism [62,63,64]. Furthermore, whole grains are rich in micronutrients (e.g., B vitamins, magnesium) and phytochemicals that contribute to glycemic control, improved insulin sensitivity, and reduced risk of metabolic disorders [65,66,67].

#### 3.2.2. Olive Oil as Primary Fat Source

Extra virgin olive oil (EVOO) is the principal source of dietary fat in the MedDiet and a defining component of its nutritional profile [68,69]. Unlike saturated fats commonly found in animal-derived products, EVOO is characterized by a high content of monounsaturated fatty acids (MUFAs), particularly oleic acid, which has been consistently associated with favorable lipid profiles, including reductions in low-density lipoprotein (LDL) cholesterol and maintenance or increases in high-density lipoprotein (HDL) cholesterol [68,70,71,72,73].

Beyond its fatty acid composition, EVOO contains a complex and biologically active matrix of minor natural compounds, particularly phenolic constituents such as hydroxytyrosol, oleuropein, tyrosol, and oleocanthal, which exhibit potent antioxidant, anti-inflammatory, and anti-atherogenic properties [74,75,76,77]. These bioactive compounds may interact with the gut microbiota, promoting the growth of beneficial microbial species and enhancing the production of metabolites with systemic health effects [78,79,80].

#### 3.2.3. Animal-Based Foods

The MedDiet is characterized by a moderate intake of animal-based foods, with a clear emphasis on fish and seafood over red and processed meats [81,82,83,84]. Fish and seafood represent key sources of high-quality protein and long-chain omega-3 polyunsaturated fatty acids (PUFAs), particularly eicosapentaenoic acid (EPA) and docosahexaenoic acid (DHA), which exert multiple cardioprotective, anti-inflammatory, and neuroprotective effects [81,82,83,84]. In addition, they provide a wide range of essential micronutrients, including vitamin D, vitamin B12, iodine, selenium, zinc, and phosphorus, as well as bioactive compounds such as taurine and marine-derived peptides, which contribute to metabolic regulation, thyroid function, antioxidant defense, and overall cardiovascular and cognitive health [81,82,83,84].

Conversely, the limited consumption of red and processed meats in the MedDiet contributes to reduced intake of saturated fatty acids, trans fatty acids, and dietary cholesterol, as well as lower exposure to potentially harmful compounds such as heme iron, advanced glycation end products (AGEs), heterocyclic amines (HCAs), polycyclic aromatic hydrocarbons (PAHs), and N-nitroso compounds, including nitrosamines, which are formed during processing and high-temperature cooking [85,86,87,88]. Processed meats may also contain high levels of sodium, preservatives (e.g., nitrates and nitrites), and oxidation products that have been associated with increased cardiometabolic and cancer risk. The substitution of red and processed meats with fish, legumes, and other plant-based protein sources is therefore considered a key mechanism underlying the health benefits of the MedDiet, contributing to improved lipid profiles, reduced inflammation, and lower overall disease risk [85,86,87,88].

Dairy products are typically consumed in moderate amounts within the MedDiet, primarily in fermented forms such as yogurt and cheese, which provide high-quality protein, calcium, phosphorus, and a broad spectrum of essential micronutrients, including vitamin B12, riboflavin (B2), vitamin A, vitamin D (in fortified products), potassium, and zinc, as well as bioactive peptides derived from milk protein fermentation [89,90,91]. Fermented dairy products are also rich in probiotics (e.g., Lactobacillus and Bifidobacterium species) and contain additional bioactive compounds such as conjugated linoleic acid (CLA), sphingolipids, and short-chain fatty acids, which may beneficially modulate the gut microbiota, enhance microbial diversity, and promote the production of metabolites with anti-inflammatory, immunomodulatory, and metabolic health-promoting effects [89,90,91].

#### 3.2.4. Alcohol Consumption

Moderate consumption of alcohol, particularly in the form of red wine during meals, is a traditional component of the MedDiet and is often embedded within social and cultural practices that emphasize conviviality and mindful eating. Red wine contains a variety of bioactive compounds, including polyphenols such as resveratrol, quercetin, and catechins, which have been associated with antioxidant, anti-inflammatory, and vasodilatory effects [92,93,94].

Importantly, the context in which wine is consumed within the MedDiet—typically in moderation, alongside meals, and within a balanced dietary and lifestyle framework—may influence its physiological effects. Importantly, current public health guidelines increasingly emphasize caution, recommending that alcohol consumption should not be initiated for health benefits and should be limited or avoided, particularly among vulnerable populations. As such, the potential benefits of wine consumption within the MedDiet should be carefully interpreted within a broader lifestyle context, and alternative sources of polyphenols, such as fruits, vegetables, and olive oil, should be prioritized [92,93,94].

#### 3.2.5. The Combined Effect of MedDiet Pattern

The combined effect of these dietary components results in a nutritionally balanced and metabolically favorable dietary pattern, characterized by: (i) High nutrient density relative to caloric intake, (ii) A favorable omega-6 to omega-3 fatty acid ratio, (iii) Low glycemic load and improved insulin sensitivity, and (iv) High antioxidant and anti-inflammatory capacity. These attributes contribute to improved metabolic homeostasis, reduced systemic inflammation, and lower risk of chronic diseases [95,96,97,98].

### 3.3. Lifestyle and Cultural Dimensions

**Figure 3:**
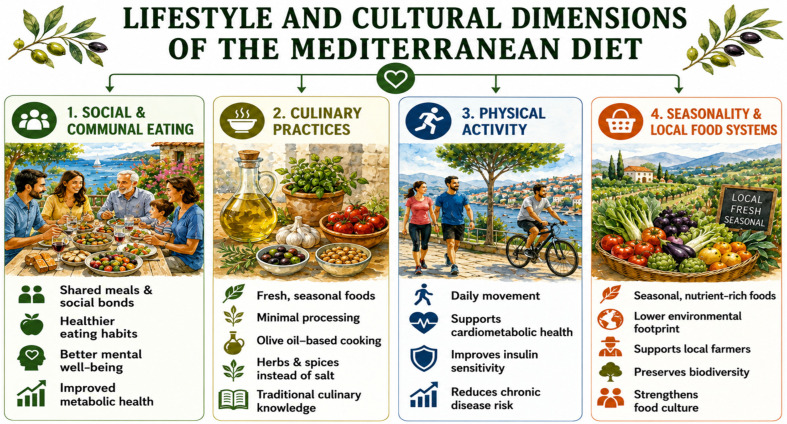
Mediterranean diet as a holistic way of life which encompasses social practices, culinary traditions, and behavioral norms.

A defining feature of the MedDiet is its integration within a broader lifestyle and cultural framework, which enhances both its health benefits and long-term sustainability [99,100]. Unlike many contemporary dietary patterns that focus primarily on nutrient composition, the MedDiet is embedded in a holistic way of life that encompasses social practices, culinary traditions, and behavioral norms as shown in Figure 3. This integrative perspective is essential for understanding not only its nutritional value but also its enduring adherence and cultural resilience.

#### 3.3.1. Social and Communal Eating

Communal eating is a central aspect of Mediterranean culture, reflecting deeply rooted traditions of family meals and social interaction [101,102,103]. These practices are embedded within a broader socio-cultural system in which food is not only a source of nutrition but also a vehicle for reinforcing interpersonal relationships, intergenerational bonding, and cultural continuity. In this context, culinary knowledge, food preparation techniques, and dietary norms are often transmitted across generations during shared meals, preserving cultural heritage and reinforcing collective identity. Regular shared meals contribute to psychological well-being by fostering a sense of belonging, emotional support, and social identity, all of which are increasingly recognized as important determinants of overall health [101,102,103].

From a nutritional and behavioral perspective, shared meals are associated with healthier eating behaviors, including slower eating pace, improved satiety signaling, enhanced appetite regulation, greater consumption of nutrient-dense foods, and better adherence to traditional dietary patterns such as the Mediterranean diet [104,105]. Communal eating may also reduce distracted or emotional eating, promote dietary variety, encourage structured meal patterns, and decrease reliance on irregular eating behaviors, thereby supporting favorable metabolic outcomes [104,105].

Beyond nutrition, social eating is positively linked to mental health, being associated with lower risks of depression, anxiety, and social isolation through mechanisms involving reduced loneliness, stronger social support, improved stress buffering, and better regulation of the hypothalamic–pituitary–adrenal (HPA) axis [106,107,108]. Shared meals may additionally foster mindfulness and emotional regulation, while in older adults they have been associated with improved cognitive function and reduced frailty risk [106,107,108]. Overall, communal eating represents a key element of the Mediterranean dietary pattern, illustrating how dietary practices are deeply intertwined with social structure and psychological well-being, thereby reinforcing the multidimensional health benefits of the Mediterranean lifestyle.

#### 3.3.2. Culinary Practices

Traditional Mediterranean culinary practices emphasize simplicity, freshness, and seasonality, with a strong focus on preserving the natural flavors, texture, and nutritional integrity of ingredients [109,110]. These practices are closely linked to the use of minimally processed foods and short food supply chains, which help preserve nutrient density and reduce exposure to additives commonly found in ultra-processed foods. In addition, reliance on locally sourced ingredients harvested at optimal ripeness contributes to higher concentrations of vitamins, minerals, and phytochemicals, thereby enhancing the overall bioactive potential of meals. Cooking methods such as grilling, baking, steaming, stewing, and sautéing with olive oil are preferred over deep frying, thereby limiting the formation of harmful compounds such as trans fatty acids and AGEs, which are associated with oxidative stress and cardiometabolic risk. These gentler cooking techniques also help maintain the bioavailability of heat-sensitive micronutrients and phytochemicals, further enhancing the nutritional quality of meals [109,110].

The Mediterranean culinary tradition integrates flavor and health through the extensive use of herbs and spices such as oregano, basil, rosemary, garlic, thyme, mint, and parsley, which enhance palatability while reducing the need for added salt and supporting lower sodium intake [111]. Beyond their culinary role, these ingredients provide bioactive compounds—including polyphenols, flavonoids, sulfur-containing compounds, and essential oils—with antioxidant, anti-inflammatory, antimicrobial, cardiometabolic, and gut microbiota-modulating properties [111].

Mediterranean culinary knowledge is traditionally transmitted across generations, promoting cultural continuity, food literacy, cooking skills, and long-term adherence to home-prepared, whole-food dietary patterns [112]. However, urbanization, time constraints, and increasing reliance on convenience foods have contributed to the erosion of these practices, threatening the preservation of Mediterranean dietary heritage and its associated health benefits [112].

#### 3.3.3. Physical Activity

Physical activity is an integral component of the Mediterranean lifestyle, historically embedded in daily routines through occupational labor, active transportation, and recreational and social activities [113,114,115]. In traditional Mediterranean societies, physical movement was rarely structured as formal exercise but rather accumulated through farming, walking, household tasks, and community engagement, resulting in sustained moderate-intensity activity patterns across the lifespan. This continuous, lifestyle-integrated activity profile contrasts with the sedentary behaviors commonly observed in modern urban populations and is considered a key determinant of the historically favorable health outcomes observed in Mediterranean regions [113,114,115].

The health benefits of the Mediterranean lifestyle arise from the synergistic interaction between a nutrient-dense dietary pattern and regular physical activity, which together improve cardiovascular health, insulin sensitivity, body composition, lipid metabolism, endothelial function, inflammation, and oxidative stress regulation [113,114,115]. Epidemiological evidence indicates that adherence to both components is associated with lower risks of all-cause mortality, cardiovascular disease, and metabolic disorders than either component alone [113,114,115].

Accordingly, the Mediterranean diet is increasingly viewed within a broader lifestyle framework that integrates physical activity as a central pillar of health promotion. This holistic approach aligns with public health recommendations emphasizing reduced sedentary behavior and regular exercise, highlighting that optimal health outcomes result from the combined effects of diet, physical activity, and other healthy behaviors [113,114,115].

#### 3.3.4. Seasonality and Local Food Systems

Seasonality is a fundamental principle of the MedDiet, reflecting a close alignment between dietary practices and natural agricultural cycles [116]. This seasonal pattern of consumption ensures that foods are typically harvested at peak ripeness, which is associated with higher concentrations of vitamins, minerals, and phytochemicals, including polyphenols and carotenoids. These compounds tend to decline during prolonged storage or long-distance transport, meaning that seasonal consumption may enhance overall nutrient density and bioactive potential of the diet [117]. In addition, seasonal eating naturally promotes dietary diversity across the year, as food choices shift according to agricultural availability, thereby contributing to a broader range of micronutrient intake and improved dietary adequacy [116,117].

From an environmental perspective, seasonal food consumption reduces reliance on greenhouse cultivation, artificial ripening, long-distance transportation, and energy-intensive cold storage systems. These reductions translate into lower GHG emissions, decreased fossil fuel consumption, and reduced food system energy demand. Moreover, seasonal diets are closely aligned with agroecological principles, supporting more efficient use of natural resources and reducing pressure on ecosystems [116,117]. Seasonal production often requires fewer inputs of irrigation water, synthetic fertilizers, and pesticides, thereby contributing to improved resource-use efficiency and reduced environmental pollution [116,117].

Local food systems play a critical role in reinforcing the sustainability of the MedDiet by shortening supply chains and strengthening regional agricultural economies [118]. By prioritizing locally produced foods, these systems contribute to economic resilience through support of smallholder farmers, preservation of rural livelihoods, and retention of value within local communities. In addition, local production systems are often associated with greater crop diversity and traditional farming practices, which contribute to the preservation of regional biodiversity and landscape heterogeneity [118]. Shorter supply chains may also reduce transportation-related emissions, packaging requirements, and post-harvest losses, thereby lowering the overall environmental footprint of food systems [118].

Beyond environmental and economic benefits, local food systems also enhance social sustainability by fostering closer relationships between producers and consumers. This increased proximity improves transparency, trust, and food awareness, while also reinforcing cultural identity and traditional food knowledge. Local markets, community-supported agriculture initiatives, and traditional food networks can further strengthen social cohesion by creating opportunities for community engagement and direct interaction among stakeholders within the food system. In addition, the preservation and transmission of traditional culinary practices across generations contribute to safeguarding cultural heritage and maintaining the distinct food culture that characterizes Mediterranean societies [116,117,118].

In summary, seasonality and localization constitute essential pillars of the MedDiet and contribute substantially to its sustainability. By promoting nutrient-rich foods, reducing environmental impacts, supporting local economies, preserving biodiversity, and strengthening cultural heritage and social cohesion, these practices exemplify the multidimensional nature of sustainable diets.

## 4. Sustainability Framework of Mediterranean Diet: Multidimensional Perspectives

**Figure 4:**
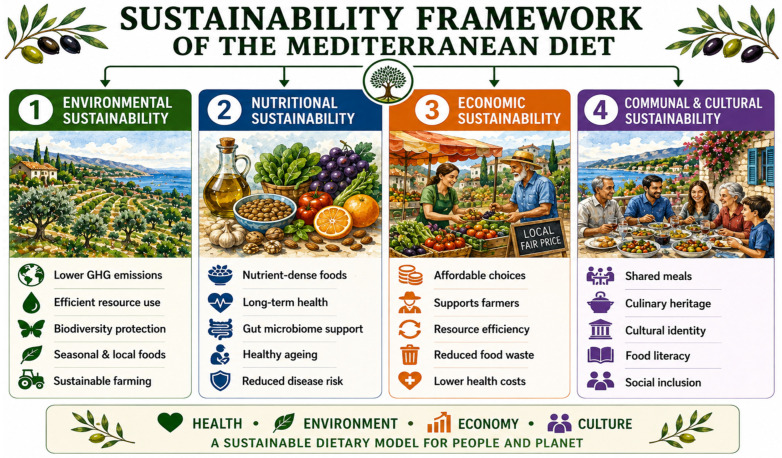
Sustainability framework of Mediterranean Diet and its multidimensional perspectives.

Evaluating modern dietary patterns requires a holistic paradigm shift from basic nutrient sufficiency to a comprehensive analysis of systemic impact. Contemporary food systems operate at the critical intersection of global ecology, public health, macroeconomics, and cultural heritage. Consequently, assessing the viability and long-term resilience of any diet necessitates a multi-dimensional framework that spans four interconnected pillars: environmental protection, nutritional adequacy, economic viability, and socio-cultural alignment as depicted in Figure 4.

Among modern dietary archetypes, the MedDiet serves as a premier benchmark across this four-part matrix. Celebrated for its clinical health outcomes and recognized globally as a vital cultural asset, the MedDiet offers a unique case study in balancing human health with planetary boundaries. The following sections critically analyze how dietary choices reverberate across these distinct yet overlapping dimensions, exploring the systemic benefits, inherent trade-offs, and structural challenges of implementing sustainable food models in an increasingly globalized world.

### 4.1. Environmental Sustainability

Environmental sustainability has become a central criterion in the evaluation of dietary patterns, reflecting the substantial contribution of food systems to GHG emissions, land degradation, freshwater depletion, biodiversity loss, and disruption of biogeochemical cycles [119,120,121,122,123]. However, sustainability is a complex and context-dependent concept influenced not only by dietary choices but also by production systems, trade networks, technological inputs, consumer behavior, and regional ecological conditions. Consequently, sustainability assessments require a systems-level perspective rather than simple comparisons between plant- and animal-based diets [119,120,121,122,123].

LCA is widely regarded as the gold standard for quantifying the environmental impacts of foods and dietary patterns, evaluating resource use and emissions throughout the food supply chain [119,120,121,124]. Common indicators include GHG emissions, land and water use, energy demand, eutrophication, acidification, and biodiversity impacts. Nevertheless, LCA results are sensitive to methodological choices, including system boundaries, allocation methods, assumptions regarding land-use change, and treatment of ecosystem services, limiting direct comparability among studies [119,120,121,124].

LCA evidence consistently shows that plant-rich dietary patterns generally have lower environmental footprints than diets high in animal-source foods [125,126]. Livestock production is associated with feed conversion losses, methane emissions, manure management, and extensive land requirements, whereas plant foods typically require fewer resources and generate lower emissions per unit of energy or protein [127,128]. However, environmental performance varies considerably within food categories. Some plant foods may be associated with intensive irrigation, fertilizer use, habitat conversion, or long-distance transportation, demonstrating that sustainability depends not only on food type but also on production and supply-chain characteristics [127,128].

Within this context, the MedDiet occupies a generally favorable position. Its emphasis on fruits, vegetables, legumes, whole grains, nuts, and olive oil, combined with moderate consumption of animal products and limited intake of red and processed meats, is associated with lower environmental impacts than most Western dietary patterns [125]. Additional sustainability attributes, including seasonality, dietary diversity, local food systems, and traditional agricultural practices, may further enhance its environmental performance [128,129]. However, increasing consumption of resource-intensive foods within Mediterranean countries may partially diminish these traditional advantages [128,129].

Water use represents another important sustainability dimension. Agriculture accounts for approximately 70% of global freshwater withdrawals, with animal-source foods generally exhibiting larger water footprints than plant-based alternatives [130,131]. Although Mediterranean crops such as olives, legumes, and cereals are often considered suitable for water-scarce environments, the expansion of irrigated agriculture and export-oriented horticultural production has increased pressure on freshwater resources in some regions [130,131]. Thus, water sustainability depends not only on dietary composition but also on agricultural practices and water-management strategies.

Environmental sustainability also encompasses biodiversity conservation and ecosystem functioning. Traditional Mediterranean agroecosystems—including mixed farming systems, crop diversification, agroforestry, olive groves, and extensive grazing systems—have historically supported biodiversity and cultural landscapes [121,132,133]. By promoting dietary diversity and local food varieties, the MedDiet may contribute to the conservation of genetic resources and agricultural heritage. Nevertheless, biodiversity outcomes remain highly dependent on land-management practices and supportive agricultural policies and cannot be guaranteed through dietary change alone [133,134].

Importantly, environmental sustainability should not be viewed as an intrinsic property of any dietary pattern. Greenhouse-grown vegetables, highly processed plant-based products, air-freighted foods, and excessive packaging can offset some of the expected benefits of plant-rich diets [134]. Conversely, moderate consumption of sustainably produced animal-source foods may, in certain contexts, have lower environmental impacts than highly industrialized plant-food production systems [134,135].

Collectively, while the MedDiet remains one of the most compelling dietary models for promoting environmental sustainability, its environmental performance should not be assumed to be universally superior. Its benefits depend on context-specific implementation, including sustainable agricultural practices, seasonal and local food consumption, efficient resource management, and effective food-system governance [134,135]. This reflects a broader shift in sustainability research from evaluating individual foods toward assessing entire food systems and their interactions with environmental, economic, and social determinants.

### 4.2. Nutritional Sustainability

Nutritional sustainability encompasses not only the adequacy of nutrient intake but also the ability of dietary patterns to support long-term health outcomes, disease prevention, and resilience across populations and generations [136,137]. This concept reflects a shift from short-term nutritional sufficiency toward a more holistic understanding of diet as a determinant of lifelong health and well-being.

A nutritionally sustainable diet must provide all essential nutrients in appropriate quantities while minimizing the risk of diet-related NCDs, including CVDs, metabolic disorders, and certain cancers. In this context, the MedDiet has consistently demonstrated strong associations with improved health outcomes, making it a model for nutritional sustainability.

Importantly, nutritional sustainability also addresses the global “double burden” of malnutrition, characterized by the coexistence of undernutrition (e.g., micronutrient deficiencies, stunting) and overnutrition (e.g., obesity, metabolic syndrome) within populations and even individuals [138,139]. This dual challenge is particularly evident in low- and middle-income countries undergoing rapid nutrition transitions, where traditional diets are increasingly replaced by energy-dense, nutrient-poor processed foods [138,139].

The MedDiet offers a potential solution to this paradox by promoting nutrient-dense, minimally processed foods that provide both macronutrient balance and micronutrient adequacy. Its high content of fruits, vegetables, whole grains, and legumes ensures sufficient intake of vitamins, minerals, and dietary fiber, while its healthy fat profile supports metabolic health [136,137].

Another critical dimension of nutritional sustainability is the interaction between diet and the gut microbiome, which plays a key role in immune function, metabolism, and disease risk. Diets rich in plant-based foods and dietary fiber—such as the MedDiet—have been shown to promote microbial diversity and the production of beneficial metabolites such as SCFAs, which contribute to systemic health [140].

Furthermore, nutritional sustainability must consider intergenerational effects, including maternal and early-life nutrition, which have lasting impacts on growth, development, and disease risk. The MedDiet, with its balanced nutrient profile and emphasis on whole foods, is well-suited to support healthy development across the life course [141,142].

Despite its advantages, challenges remain in ensuring equitable access to nutritionally sustainable diets. Socioeconomic disparities, food environments, and cultural factors can all influence dietary choices, highlighting the need for integrated policy approaches that address both supply and demand.

### 4.3. Economic Sustainability

Economic sustainability is a fundamental yet often underexplored component of sustainable diets, encompassing affordability, accessibility, economic resilience, and the long-term viability of food systems for both consumers and producers [143,144]. Beyond food prices, it includes broader issues such as income inequality, market structures, trade policies, food-system resilience, and the distribution of costs and benefits across stakeholders [143,144].

Affordability remains a major barrier to healthy eating. Nutrient-dense foods such as fruits, vegetables, nuts, fish, and minimally processed products often have higher per-calorie costs than energy-dense ultra-processed foods rich in refined carbohydrates, added sugars, and unhealthy fats [145,146]. This disparity is partly explained by market prices that rarely reflect the true societal costs of food production, including environmental degradation, healthcare expenditures, and other social externalities [145,146]. Consequently, less nutritious foods may appear economically advantageous despite generating substantial long-term societal costs.

The economic accessibility of the MedDiet remains debated [147,148]. While some studies report higher household food expenditures, others suggest that a traditional Mediterranean dietary pattern centered on legumes, whole grains, seasonal produce, and limited meat consumption can be cost-neutral or even cost-saving [147,148]. Affordability varies considerably according to geographic location, income level, food environment, cultural preferences, and market conditions. As a result, socioeconomic inequalities may limit access to key Mediterranean foods and constrain large-scale adoption [147,148].

Importantly, economic evaluations often focus on direct food costs while overlooking longer-term benefits. Greater adherence to healthy dietary patterns, including the MedDiet, may reduce healthcare expenditures by lowering the incidence of obesity, cardiovascular disease, type 2 diabetes, and other chronic conditions [148,149]. However, these long-term societal savings may not offset the immediate financial constraints faced by many households, highlighting a persistent tension between individual affordability and societal cost-effectiveness [148,149].

Economic sustainability also depends on the viability of food production systems. Small-scale and traditional agricultural systems, often central to Mediterranean food culture, face increasing pressures from globalization, industrialized agriculture, volatile commodity markets, and rising production costs [143,149,150]. Although these systems may generate environmental and socio-cultural benefits, their long-term viability frequently depends on supportive policies, infrastructure investments, and market incentives [143,149,150].

Food pricing policies, subsidies, and taxation strongly influence dietary behaviors and food-system outcomes [145,151,152]. In many regions, agricultural subsidies continue to favor commodity crops and livestock production while providing comparatively limited support for fruits, vegetables, legumes, and other foods central to sustainable diets. Consequently, fiscal incentives for healthy foods, taxation of unhealthy products, and subsidy reforms have been proposed to better align economic incentives with public health and sustainability goals. Nevertheless, their effectiveness and equity implications remain subjects of ongoing debate [145,151,152].

Food waste represents another important source of economic inefficiency, resulting in the loss of both edible food and the resources embedded throughout production, processing, transportation, and retail distribution [153,154]. Within dietary patterns such as the MedDiet, which emphasize fresh and seasonal foods, waste reduction is particularly relevant. Traditional Mediterranean practices, including food preservation and creative reuse of leftovers, may contribute to improved resource efficiency and lower economic losses [153,154].

Despite growing interest in the economics of sustainable diets, important knowledge gaps remain. Many analyses fail to fully account for environmental externalities, ecosystem services, public health benefits, labor conditions, and social inequalities, potentially underestimating the true economic value of sustainable dietary patterns [145,146,150,153]. Furthermore, differences in methodology, geographic context, and consumer behavior assumptions limit comparability across studies.

Ultimately, the economic sustainability of the MedDiet should not be viewed as an intrinsic property of the dietary pattern itself, but rather as an outcome of broader food-system conditions. Achieving economic sustainability requires integrated approaches that align agricultural policy, food pricing, market structures, labor conditions, consumer behavior, and public health objectives to ensure that healthy and sustainable foods remain accessible, affordable, and economically viable for both consumers and producers.

### 4.4. Communal and Cultural Sustainability

Communal and cultural sustainability reflects the extent to which dietary patterns are culturally acceptable, socially meaningful, equitable, and aligned with local traditions and values [155]. As a key dimension of sustainable diets, it recognizes that long-term adherence depends not only on nutritional quality and environmental sustainability but also on compatibility with cultural practices, community norms, and everyday lifestyles [155].

The MedDiet is widely considered a model of communal–cultural sustainability due to its deep roots in regional traditions, culinary practices, and community structures. Recognized by UNESCO as an Intangible Cultural Heritage of Humanity, it encompasses not only specific foods but also the knowledge, skills, rituals, and social practices associated with food production, preparation, and consumption [155,156]. This broader perspective highlights the MedDiet as a cultural system that integrates food, social interaction, physical activity, and community engagement.

Traditional Mediterranean practices—including shared meals, seasonal cooking, family-centered dining, and the use of local ingredients—reinforce cultural identity, social cohesion, and intergenerational transmission of culinary knowledge [157,158]. These practices have been associated with stronger social networks, improved mental well-being, and enhanced quality of life, suggesting that the benefits of the MedDiet extend beyond nutritional composition alone [157,158].

However, the communal–cultural sustainability of the MedDiet should not be idealized. Globalization, urbanization, migration, changing household structures, and evolving labor patterns have transformed food environments, even within Mediterranean countries [159,160]. The growing availability of ultra-processed foods, reduced time for food preparation, and declining family meal traditions have contributed to lower adherence, particularly among younger generations [159,160]. As a result, the MedDiet increasingly represents a cultural ideal rather than a consistently practiced reality.

Moreover, traditional food cultures are dynamic rather than static systems. Sustainability strategies focused solely on restoring historical dietary patterns risk overlooking contemporary social realities and romanticizing the past [161]. Effective approaches must balance the preservation of beneficial cultural practices with adaptation to modern lifestyles, technological change, and evolving consumer preferences [161].

Education and food-literacy initiatives can help preserve and revitalize traditional dietary practices by promoting culinary skills, gardening, and nutrition education from an early age [162]. Nevertheless, educational interventions alone are unlikely to reverse dietary transitions without complementary policies addressing food marketing, urban food environments, and socioeconomic barriers to healthy eating [162].

Communal-cultural sustainability is also closely linked to equity and inclusivity. Access to healthy and culturally appropriate foods remains unevenly distributed across populations, reflecting disparities in income, education, geography, and food environments [163]. Consequently, dietary recommendations that fail to account for socioeconomic constraints may inadvertently widen health inequalities [163].

The transferability of the MedDiet beyond Mediterranean regions presents an additional challenge. Differences in food availability, culinary traditions, agricultural systems, and cultural preferences may limit direct adoption [159,160,161]. Increasingly, researchers argue that the broader principles underlying the MedDiet—such as plant-forward eating, seasonality, culinary traditions, and communal connectedness—are more transferable than specific foods [160,161,162,163]. This perspective supports the development of locally adapted dietary models that share common sustainability principles while respecting cultural diversity.

Therefore, communal–cultural sustainability should be viewed as a dynamic and context-dependent process rather than a fixed characteristic of any dietary pattern. In this regard, the MedDiet serves not only as a dietary model but also as a broader framework illustrating how culturally embedded food systems can support health, environmental sustainability, social cohesion, and human well-being. Its continued relevance will depend on the ability of societies to adapt its core principles to changing social, economic, and cultural conditions while preserving the values that have historically underpinned its sustainability.

## 5. Exploring the Environmental Impacts of the Mediterranean Diet: Opportunities and Challenges

The environmental sustainability of dietary patterns has become an increasingly important area of research in the context of climate change, resource depletion, and ecosystem degradation. Among various dietary models, the MedDiet has gained attention not only for its well-established health benefits but also for its potential to reduce environmental pressures associated with food systems. This section critically explores the environmental impacts of the MedDiet across four key dimensions: GHG emissions, land use and agricultural efficiency, water use and resource management, and biodiversity conservation as summarized in Table 1. By examining these interconnected aspects, the MedDiet emerges as a holistic approach that aligns human nutrition with environmental sustainability, while also highlighting the importance of production practices, resource management, and policy frameworks in maximizing its ecological benefits.

### 5.1. Greenhouse Gas (GHG) Emissions and Climate Change Mitigation

Food systems contribute approximately one-quarter to one-third of global anthropogenic GHG emissions through agricultural production, land-use change, processing, transportation, retail activities, and food waste management [164,165]. Consequently, dietary patterns have emerged as important targets for climate-change mitigation, although their environmental impacts depend on both food choices and the broader systems through which food is produced and distributed.

The MedDiet, characterized by high consumption of plant-based foods and limited intake of red and processed meats, is generally associated with lower GHG emissions than Western dietary patterns [127,166]. A major contributor to this advantage is reduced reliance on ruminant livestock, which generates substantial methane emissions through enteric fermentation and manure management [127,166]. In contrast, legumes, cereals, fruits, and vegetables typically exhibit lower emissions and greater resource-use efficiency due to the absence of enteric methane production and lower land requirements [127,166].

Additional environmental benefits may arise from the MedDiet’s emphasis on seasonal and locally sourced foods, potentially reducing emissions associated with storage and refrigeration [167,168]. However, evidence suggests that transportation often accounts for a relatively small proportion of total food-system emissions, whereas production practices are typically the dominant determinant of environmental impact [128,132,134]. Consequently, locally produced foods are not necessarily more climate-friendly than imported alternatives if they are produced using resource-intensive methods.

Modeling studies estimate that replacing Western dietary patterns with Mediterranean-style diets could reduce food-related GHG emissions by approximately 30–50%, depending on dietary composition, adherence levels, and regional context [167,168]. However, these estimates often rely on idealized assumptions and may not fully account for socioeconomic, cultural, and behavioral barriers that influence real-world adoption [125,127,130].

Importantly, the climate benefits of the MedDiet are not universally guaranteed. Environmental outcomes vary considerably according to production systems, supply-chain characteristics, and food choices within Mediterranean dietary patterns themselves [128,129,130,167,168]. For example, greenhouse-grown vegetables, irrigated horticultural crops, air-freighted foods, and highly processed plant-based products may generate substantially higher emissions than minimally processed local foods. Similarly, the sustainability of fish consumption depends heavily on fisheries management, stock status, fishing methods, feed sources, and aquaculture practices, with overfishing and poorly managed aquaculture potentially undermining environmental benefits [128,129,130,165,168].

Furthermore, GHG emissions represent only one dimension of environmental sustainability. Foods with relatively low carbon footprints may still exert substantial pressures on freshwater resources, biodiversity, land use, nutrient cycles, and ecosystem integrity [122,123,125,127,129,168]. Consequently, evaluating dietary sustainability solely through a carbon lens may oversimplify important trade-offs among environmental objectives.

Interpretation of the evidence also requires caution because LCA studies exhibit considerable methodological variability, including differences in system boundaries, allocation methods, treatment of land-use change, and assumptions regarding production practices [166,167]. These factors can significantly influence estimated emissions and complicate direct comparisons across studies.

Moreover, dietary change alone is unlikely to achieve meaningful climate mitigation without parallel improvements in food-system practices. Sustainable agriculture, renewable energy adoption, reductions in food loss and waste, supply-chain efficiencies, and policies addressing overconsumption may contribute as much as—or potentially more than—dietary shifts alone [131,134,167]. Likewise, reducing excessive caloric intake can substantially lower food-related emissions regardless of dietary pattern [166,167,168].

Overall, the MedDiet represents one of the most promising dietary frameworks for reducing food-related GHG emissions and supporting climate goals. However, its environmental advantages should not be viewed as inherent or universally applicable. Rather, they depend on context-specific factors including production methods, resource management, food waste, supply-chain efficiency, and broader food-system governance. This perspective highlights the growing recognition that climate-compatible diets require both dietary change and systemic transformation of food production, distribution, and consumption.

### 5.2. Land Use and Agricultural Efficiency

Land use is a critical component of environmental sustainability, as agriculture occupies nearly 50% of the world’s habitable land and is a major driver of deforestation, habitat loss, and ecosystem degradation [169,170]. Beyond the area occupied, sustainability depends on land-management practices, biodiversity impacts, soil health, and ecosystem services. Land-use change is also a significant source of GHG emissions through the conversion of forests, grasslands, and other natural ecosystems into agricultural land [169,170].

The MedDiet promotes more land-efficient food production through its emphasis on plant-based foods and lower consumption of animal products, particularly ruminant meat [169,170]. Livestock production requires extensive grazing land and feed crops, resulting in substantially greater land demands and lower feed-to-food conversion efficiency than plant-based foods. In contrast, legumes and cereals generally provide higher caloric and protein yields per hectare and can enhance soil fertility through nitrogen fixation, reduced fertilizer requirements, and improved crop rotations [171,172,173].

Traditional Mediterranean agricultural systems further support sustainable land use through diversified practices such as crop rotation, intercropping, agroforestry, and mixed crop–livestock systems, which improve soil quality, reduce erosion, and enhance resilience to climate variability [171,172,173]. However, agricultural intensification and specialization in many Mediterranean regions have reduced some of these ecological benefits, highlighting a gap between the theoretical sustainability of the MedDiet and modern production realities [171,172,173].

Reduced land demand associated with Mediterranean-style diets may alleviate pressure on forests, grasslands, and other ecosystems, potentially supporting biodiversity conservation, carbon sequestration, and ecological restoration [171,172,173]. Nevertheless, land sparing does not automatically lead to environmental recovery, as freed land may be redirected toward urban development, bioenergy production, or other economic uses [174,175]. Consequently, dietary shifts alone are insufficient without supportive land-use planning and environmental governance.

Critically, lower land requirements do not necessarily guarantee greater sustainability. Intensive cultivation of plant-based crops may still contribute to soil degradation, water depletion, habitat loss, and biodiversity decline when based on monocultures or unsustainable management practices [174,175]. Conversely, some extensive grazing systems can support biodiversity, maintain semi-natural habitats, and preserve traditional cultural landscapes. These examples illustrate that simple comparisons between plant- and animal-based foods may overlook important ecological trade-offs.

Hence, the environmental benefits of the Mediterranean diet are greatest when dietary shifts are combined with sustainable agricultural practices, biodiversity conservation, regenerative farming, and effective land-management policies. Consequently, sustainability assessments should consider not only land-use efficiency but also ecological integrity, soil health, biodiversity, and ecosystem services. Thus, the long-term sustainability of Mediterranean dietary patterns depends on coordinated changes in dietary behavior, agricultural production, and land-governance systems.

### 5.3. Water Use and Resource Efficiency

Water scarcity is a growing global challenge, with agriculture accounting for approximately 70% of freshwater withdrawals worldwide [176,177]. Increasing population growth, climate change, urbanization, and demand for water-intensive foods are placing additional pressure on limited freshwater resources. Water footprints—comprising blue, green, and grey water—are commonly used to assess food-related water use, although their interpretation should consider local hydrological conditions, ecosystem vulnerability, and regional water scarcity [176,177].

Overall, plant-based foods generally require less water than animal-source products, particularly when accounting for feed production, animal husbandry, processing, and supply chains. Consequently, the MedDiet, characterized by high consumption of plant foods and relatively low intake of red meat, is often associated with a lower water footprint than Western dietary patterns [177,178]. However, water requirements vary substantially according to crop type, irrigation practices, climatic conditions, and production systems, making broad comparisons between food categories overly simplistic [178,179].

The relationship between the MedDiet and water sustainability is therefore more complex than often assumed. Several staple Mediterranean foods, including nuts, fruits, vegetables, and olive oil, may have substantial water requirements, particularly when produced in water-stressed regions or under intensive irrigation systems [176,178]. Consequently, water sustainability depends not only on dietary composition but also on production methods, regional resource availability, and agricultural management practices.

Notably, water-use efficiency does not necessarily equate to sustainable water use. Agricultural production can generate environmental pressures when it relies on overexploited aquifers, disrupts aquatic ecosystems, or competes with other societal water needs. Conversely, water-intensive crops may be sustainably produced in regions with abundant renewable water resources [176,177,178]. For this reason, water scarcity footprints and water stress-adjusted indicators are increasingly used to provide more context-sensitive assessments of agricultural water use [176,177,178].

Sustainable water management is therefore essential for the long-term viability of Mediterranean food systems. Strategies such as precision irrigation, rainwater harvesting, drought-tolerant crop varieties, conservation agriculture, and improved soil management can enhance water-use efficiency while maintaining productivity [179,180]. Traditional Mediterranean farming systems, often based on rain-fed agriculture and diversified cropping, provide valuable examples of adaptation to water-limited environments, although their scalability under future climate and food-demand pressures remains uncertain [179,180].

Water sustainability is also influenced by global trade. The importation of water-intensive products from regions with greater water availability may alleviate local water scarcity but can simultaneously transfer environmental pressures to exporting countries through “virtual water” flows [180,181]. Likewise, food waste represents a significant loss of embedded water resources, making waste reduction an important strategy for improving water-use efficiency, particularly in diets rich in fresh and perishable foods such as the MedDiet [180,181].

Climate change is expected to intensify water scarcity through altered precipitation patterns, increased drought frequency, and greater hydrological variability, especially in Mediterranean regions already considered climate-change hotspots [180,181]. These trends may affect the sustainability of traditional Mediterranean crops and require adaptations in both agricultural production systems and dietary recommendations.

Policy measures—including water-pricing mechanisms, groundwater regulation, investment in water-efficient infrastructure, and incentives for drought-resilient agriculture—are increasingly recognized as critical components of sustainable water governance [179,181]. Advances in precision agriculture, remote sensing, and digital irrigation technologies can further improve water-use efficiency, although technological solutions must be accompanied by broader reforms in agricultural policy, consumption patterns, and resource management.

Collectively, the Mediterranean diet generally has a lower water footprint than Western dietary patterns because of its reduced dependence on livestock production. However, its water sustainability depends on dietary choices, agricultural practices, climate, regional water availability, trade, and governance. Therefore, water sustainability should be assessed using a systems-based approach that considers both water use and its ecological, social, and economic impacts.

### 5.4. Biodiversity Conservation

Biodiversity underpins essential ecosystem services, including pollination, nutrient cycling, soil fertility, pest regulation, climate regulation, and ecosystem resilience. However, modern food systems are both dependent on and major drivers of biodiversity loss through habitat conversion, intensive livestock production, monocultures, and widespread agrochemical use [182,183]. Consequently, biodiversity conservation has become a central component of sustainable food-system transformation.

The MedDiet is often regarded as a biodiversity-friendly dietary pattern because of its emphasis on dietary diversity, traditional foods, and locally adapted crops [184,185]. By promoting a wide range of fruits, vegetables, legumes, cereals, nuts, herbs, and other plant foods, it may support agricultural biodiversity and reduce dependence on a limited number of globally dominant commodities [183,184,185]. Traditional Mediterranean farming systems also preserve landrace varieties and indigenous animal breeds, which contribute to genetic diversity and enhance the adaptive capacity of agricultural systems under changing environmental conditions [184,185].

Mediterranean agroecosystems have historically incorporated diversified practices such as crop rotations, agroforestry, mixed farming systems, olive groves, and terraced landscapes, creating heterogeneous habitats that support pollinators, beneficial insects, birds, and soil microorganisms [186,187]. However, agricultural intensification, monoculture expansion, greenhouse production, and intensive irrigation have increasingly threatened these biodiversity benefits in some Mediterranean regions [186,187].

The MedDiet’s lower reliance on intensive livestock production may also reduce pressures associated with deforestation, habitat conversion, and nutrient pollution [186,187]. Nevertheless, biodiversity outcomes cannot be attributed solely to dietary composition. Some plant-based production systems may contribute to habitat degradation, pesticide use, water depletion, and monoculture expansion, whereas certain extensive livestock systems can help maintain semi-natural habitats and traditional cultural landscapes [184,185,186,187]. Therefore, biodiversity impacts depend largely on agricultural management practices, land-use decisions, and governance frameworks.

Assessing biodiversity remains challenging because it encompasses multiple dimensions, including species richness, genetic diversity, ecosystem integrity, and functional diversity, which cannot be captured by a single indicator [182,183]. Furthermore, globalization, agricultural intensification, and the growing demand for standardized commodities continue to threaten both biological and cultural diversity, reducing many of the characteristics that historically supported Mediterranean food systems [183,185,188].

Importantly, biodiversity conservation is not only an environmental objective but also a prerequisite for long-term food-system resilience [187,188]. Diverse agricultural systems are generally more resistant to climate change, pest outbreaks, soil degradation, and water scarcity, while consumer demand for diverse local foods can provide incentives for maintaining traditional varieties and biodiversity-friendly farming practices [187,188].

However, dietary change alone is insufficient to halt biodiversity loss. Realizing the biodiversity potential of the MedDiet requires supportive agricultural policies, protection of genetic resources, agroecological farming practices, landscape-scale conservation strategies, and stronger food-system governance [188,189]. Thus, the MedDiet should be viewed not only as a dietary model but also as a broader framework integrating dietary diversity, cultural heritage, agricultural biodiversity, and ecosystem stewardship within sustainable food systems.

## 6. Health Impacts of the Mediterranean Diet

The MedDiet is one of the most extensively studied and health-promoting dietary patterns, supported by strong nutritional, clinical, and epidemiological evidence. Beyond its cultural significance, it provides a nutrient-rich and balanced framework for health promotion, disease prevention, and long-term well-being. Its benefits are mediated through a favorable nutritional profile, protection against major chronic diseases, and multiple underlying biological mechanisms, highlighting the MedDiet as a holistic, evidence-based approach to lifelong health.

### 6.1. Nutritional Profile

The MedDiet is widely regarded as a nutritionally optimal dietary pattern, characterized by a balanced intake of macronutrients and a high density of essential micronutrients and bioactive compounds [190]. Unlike reductionist dietary models that focus on single nutrients, the MedDiet emphasizes dietary quality, diversity, and synergy, which collectively support optimal physiological function and long-term health.

From a macronutrient perspective, the MedDiet typically provides:

- Moderate total fat intake (approximately 30–40% of total energy), primarily from monounsaturated fatty acids (MUFAs) derived from olive oil.
- Adequate protein intake, largely from plant-based sources (legumes, nuts, seeds) and complemented by moderate consumption of fish and dairy.
- Carbohydrates predominantly from whole grains, fruits, and vegetables, resulting in a low glycemic load.

This macronutrient distribution contributes to improved metabolic regulation, including enhanced insulin sensitivity and glycemic control [190].

The micronutrient profile of the MedDiet is equally notable. High consumption of fruits and vegetables ensures adequate intake of vitamins (e.g., vitamin C, folate), minerals (e.g., potassium, magnesium), and a wide spectrum of phytochemicals, including polyphenols, flavonoids, and carotenoids. These compounds exhibit antioxidant and anti-inflammatory properties, which play a critical role in disease prevention [191].

Dietary fiber intake is another defining feature, often exceeding recommended levels due to the emphasis on whole plant foods. Fiber contributes to satiety, supports gastrointestinal health, and promotes favorable lipid and glucose metabolism. Moreover, the high fiber content of the MedDiet fosters a diverse and metabolically active gut microbiota, which is increasingly recognized as a key determinant of health [192].

In addition, the MedDiet is characterized by a favorable fatty acid profile, with a low ratio of saturated to unsaturated fats and an improved balance between omega-6 and omega-3 fatty acids. This balance is essential for regulating inflammatory processes and maintaining cardiovascular health [193].

Importantly, the nutritional adequacy of the MedDiet extends across the life course, making it suitable for diverse populations, including children, adults, and older individuals. Its flexibility and adaptability further enhance its relevance in different cultural and geographic contexts [194].

### 6.2. Chronic Disease Prevention

A substantial body of epidemiological and clinical evidence supports the role of the Mediterranean diet in the prevention and management of major chronic diseases, including CVD, type 2 diabetes mellitus, obesity, and certain types of cancer [195]. These findings have positioned the MedDiet as one of the most extensively studied and consistently supported dietary patterns in nutrition science.

#### 6.2.1. Cardiovascular Disease

The strongest evidence relates to cardiovascular health. Prospective cohort studies and randomized controlled trials have demonstrated that high adherence to the MedDiet is associated with significant reductions in cardiovascular morbidity and mortality [24,196]. The landmark PREDIMED trial provided robust experimental evidence, showing that individuals following a MedDiet supplemented with extra virgin olive oil or nuts experienced a substantial reduction in major cardiovascular events compared to those on a low-fat diet [22,24].

These protective effects are mediated through multiple pathways, including improvements in lipid profiles (e.g., increased HDL cholesterol, reduced LDL oxidation), blood pressure regulation, endothelial function, and reduced systemic inflammation [22,24].

#### 6.2.2. Type 2 Diabetes and Metabolic Disorders

The MedDiet has also been shown to reduce the risk of type 2 diabetes and improve glycemic control in individuals with existing disease [196,197]. Its low glycemic load, high fiber content, and healthy fat composition contribute to improved insulin sensitivity and reduced postprandial glucose excursions.

In addition, adherence to the MedDiet is associated with lower prevalence of metabolic syndrome, a cluster of risk factors including central obesity, hypertension, dyslipidemia, and insulin resistance [196,197]. These effects are particularly relevant in the context of the global rise in obesity and related metabolic disorders.

#### 6.2.3. Cancer

Emerging evidence suggests that the MedDiet may also play a role in cancer prevention, particularly for cancers of the digestive tract, breast, and prostate [198,199]. The high intake of antioxidants and anti-inflammatory compounds, combined with reduced consumption of red and processed meats, may contribute to reduced carcinogenesis. Additionally, the synergistic interactions among dietary fiber, polyphenols, and gut microbiota may contribute to the production of bioactive metabolites with anti-carcinogenic properties, further supporting its protective role [198,199].

#### 6.2.4. Other Health Outcomes

Beyond these major disease categories, the MedDiet has been associated with a range of additional health benefits, including improved cognitive function, reduced risk of neurodegenerative diseases, and enhanced mental health [195,196,197,198,199]. Growing evidence suggests that adherence to the MedDiet is linked to slower cognitive decline, improved memory performance, and a lower incidence of conditions such as Alzheimer’s disease and Parkinson’s disease, potentially through mechanisms involving reduced neuroinflammation, oxidative stress, and vascular dysfunction [195,196,197,198,199].

Furthermore, its positive effects on mental health—including reduced risk of depression and anxiety—may be mediated by improved nutrient intake (e.g., omega-3 fatty acids, B vitamins, and polyphenols), as well as beneficial modulation of the gut–brain axis via changes in the gut microbiota [195,196,197,198,199]. These findings highlight the broad and systemic impact of dietary patterns on human health, extending beyond physical health to encompass cognitive and psychological well-being across the lifespan. Importantly, the consistency of evidence across observational studies, clinical trials, and meta-analyses strengthens the causal inference between MedDiet adherence and improved health outcomes [195,196,197,198,199].

### 6.3. Mechanisms of Action

The health benefits of the MedDiet are mediated through a complex network of biological and molecular mechanisms, reflecting the synergistic interactions among its diverse components [200,201,202,203,204,205]. These mechanisms operate across multiple physiological systems, including metabolic, inflammatory, and microbial pathways. The most important and well-characterized molecular mechanisms of action of MedDiet underlying its health benefits are summarized in Table 2.

#### 6.3.1. Anti-Inflammatory Effects

Chronic low-grade inflammation is a key driver of many non-communicable diseases. The MedDiet exerts potent anti-inflammatory effects, largely due to its high content of bioactive compounds such as polyphenols, omega-3 fatty acids, dietary fiber, vitamins (e.g., C and E), and minerals with antioxidant properties [200,201]. In addition, the overall dietary pattern—characterized by low intake of refined carbohydrates, trans fats, and ultra-processed foods—helps attenuate postprandial inflammatory responses and oxidative stress, which are key contributors to chronic inflammation [200,201].

Polyphenols found in olive oil, fruits, vegetables, and wine modulate inflammatory signaling pathways, including the inhibition of nuclear factor kappa B (NF-κB), a central regulator of inflammation. This results in reduced production of pro-inflammatory cytokines such as interleukin-6 (IL-6) and tumor necrosis factor-alpha (TNF-α), alongside improved immune regulation [200,201]. Furthermore, omega-3 fatty acids derived from fish and seafood play a critical role in the resolution phase of inflammation by giving rise to specialized pro-resolving mediators, including resolvins, protectins, and maresins, which actively suppress inflammatory processes and promote tissue repair. Dietary fiber contributes to these effects through fermentation by the gut microbiota, leading to the production of SCFAs, particularly butyrate, which inhibit histone deacetylases and regulate gene expression involved in inflammatory pathways. Collectively, these mechanisms illustrate the multi-targeted and synergistic anti-inflammatory actions of the MedDiet at molecular, cellular, and systemic levels [200,201].

#### 6.3.2. Lipid Metabolism and Cardiovascular Function

The favorable fatty acid profile of the MedDiet plays a critical role in lipid metabolism and cardiovascular health [202]. Monounsaturated fats from olive oil improve lipid profiles by increasing HDL cholesterol and reducing LDL oxidation, while also enhancing LDL particle size and stability, thereby decreasing their atherogenic potential. In parallel, these fats modulate hepatic lipid metabolism by downregulating de novo lipogenesis and improving lipid clearance from circulation [202].

Omega-3 fatty acids from fish further contribute to anti-inflammatory and anti-thrombotic effects by reducing triglyceride levels, inhibiting platelet aggregation, and modulating eicosanoid synthesis toward less pro-inflammatory mediators [202]. They also influence membrane fluidity and receptor function, which can improve cellular signaling and cardiovascular responsiveness. Additionally, bioactive compounds such as polyphenols from olive oil and plant foods reduce oxidative stress and prevent the formation of oxidized LDL, a key trigger in atherogenesis [202].

In addition, the MedDiet enhances endothelial function and vascular integrity, reducing the risk of atherosclerosis and related cardiovascular events [203]. This effect is mediated through increased nitric oxide (NO) bioavailability, improved vasodilation, and reduced expression of adhesion molecules that facilitate leukocyte infiltration into the vascular wall. Furthermore, dietary fiber and plant sterols contribute to cholesterol lowering by inhibiting intestinal absorption of cholesterol and bile acids, while the overall dietary pattern supports blood pressure regulation through improved vascular tone and reduced systemic inflammation [203].

#### 6.3.3. Glycemic Control and Insulin Sensitivity

The combination of low glycemic load carbohydrates, healthy fats, and fiber contributes to improved glycemic control and insulin sensitivity [196,197]. These effects are mediated through slower digestion and absorption of carbohydrates, reduced postprandial glucose spikes, and improved hormonal regulation, including more stable insulin secretion and enhanced incretin responses (e.g., glucagon-like peptide-1, GLP-1) [196,197]. The presence of dietary fiber—particularly soluble and viscous types—delays gastric emptying and forms gel-like matrices in the intestine, further attenuating glucose absorption and improving glycemic excursions [196,197].

At the molecular level, the MedDiet influences key pathways involved in glucose metabolism and insulin signaling. Monounsaturated fatty acids from olive oil improve insulin sensitivity by enhancing cell membrane fluidity and facilitating insulin receptor function, while reducing lipotoxicity in peripheral tissues. Polyphenols from plant-based foods modulate glucose metabolism by inhibiting carbohydrate-digesting enzymes (e.g., α-amylase and α-glucosidase), reducing intestinal glucose uptake, and activating intracellular signaling pathways such as AMP-activated protein kinase (AMPK), which promotes glucose uptake and mitochondrial function [196,197].

Additionally, fermentation of dietary fiber by the gut microbiota leads to the production of SCFAs, particularly acetate and propionate, which regulate glucose homeostasis through effects on hepatic gluconeogenesis and peripheral insulin sensitivity. These metabolites also interact with G-protein-coupled receptors involved in energy balance and metabolic regulation [196,197].

Collectively, these mechanisms highlight the integrative role of the MedDiet in modulating glycemic control through coordinated effects on digestion, hormonal signaling, cellular metabolism, and gut microbiota composition.

#### 6.3.4. Gut Microbiota Modulation

One of the most rapidly evolving areas of research concerns the interaction between the MedDiet and the gut microbiota [204,205,206]. The high intake of dietary fiber and polyphenols promotes microbial diversity and the growth of beneficial bacterial species, including genera such as Bifidobacterium, Lactobacillus, and Faecalibacterium, which are associated with anti-inflammatory and metabolically favorable profiles. In addition, polyphenols undergo biotransformation by gut microbes into bioactive metabolites with enhanced bioavailability, further amplifying their systemic effects [204,205,206].

These microbes produce metabolites such as SCFAs, which have been shown to: (a) regulate immune function, (b) improve gut barrier integrity, (c) influence energy metabolism, and (d) reduce systemic inflammation. Mechanistically, SCFAs—particularly butyrate—serve as an energy source for colonocytes, enhance tight junction protein expression, and inhibit histone deacetylases, thereby modulating gene expression involved in inflammation and metabolism. They also activate G-protein-coupled receptors (e.g., GPR41 and GPR43), which play a role in appetite regulation, glucose homeostasis, and lipid metabolism [204,205,206].

The modulation of the gut microbiome represents a key mechanism through which the MedDiet exerts its systemic health effects [204,205,206]. Furthermore, the MedDiet reduces the abundance of potentially pathogenic bacteria linked to dysbiosis and endotoxemia, thereby lowering circulating lipopolysaccharide (LPS) levels and attenuating metabolic inflammation. This bidirectional interaction between diet and microbiota underscores the importance of long-term dietary patterns in shaping a resilient and functionally beneficial gut ecosystem [204,205,206].

#### 6.3.5. Anticancer Mechanisms

The anticancer effects of the MedDiet are largely attributed to its ability to modulate key biological mechanisms involved in tumor initiation, promotion, and progression. This dietary pattern is rich in polyphenols (e.g., from olive oil, fruits, and vegetables), which exert strong antioxidant activity, neutralizing ROS and thereby reducing DNA damage and mutagenesis [57,61]. Additionally, many MedDiet components display anti-inflammatory properties by downregulating pro-inflammatory cytokines (such as TNF-α and IL-6) and inhibiting signaling pathways like NF-κB, which are often overactivated in cancer [57,61].

The MedDiet also promotes apoptosis (programmed cell death) and inhibits uncontrolled cell proliferation through bioactive compounds such as resveratrol, flavonoids, and carotenoids [198,199]. Furthermore, omega-3 fatty acids from fish contribute to the suppression of tumor growth by modulating eicosanoid production and reducing angiogenesis. Another important mechanism involves the regulation of the gut microbiota; high fiber intake enhances the production of short-chain fatty acids like butyrate, which have protective effects against colorectal carcinogenesis. Finally, the MedDiet may influence epigenetic modifications, including DNA methylation and histone acetylation, thereby regulating the expression of genes involved in cancer development [198,199]. Together, these mechanisms highlight the multifaceted role of the MedDiet in cancer prevention and control.

Moreover, adherence to the MedDiet has been associated with improved immune surveillance, enhancing the activity of natural killer cells and cytotoxic T lymphocytes that target malignant cells [57,198]. Certain bioactive compounds, such as oleocanthal in extra virgin olive oil, have demonstrated the ability to inhibit cancer cell migration and metastasis. The diet also contributes to hormonal regulation, particularly by reducing circulating estrogen levels, which is relevant for hormone-dependent cancers such as breast cancer. In addition, its low glycemic load helps regulate insulin and insulin-like growth factor-1 (IGF-1) signaling, pathways that are often implicated in cancer cell growth and survival. Lastly, the synergistic interaction among various nutrients and phytochemicals within the MedDiet enhances their overall anticancer efficacy beyond the effect of individual components [73,199].

#### 6.3.6. Emerging Mechanisms

Recent research has also highlighted additional mechanisms, including:

(a) Epigenetic regulation, whereby dietary components influence gene expression without altering DNA sequence, through mechanisms such as DNA methylation, histone modification, and microRNA expression. Bioactive compounds in the MedDiet—particularly polyphenols from olive oil, fruits, vegetables, and wine—have been shown to modulate epigenetic enzymes such as DNA methyltransferases and histone deacetylases, thereby influencing the expression of genes involved in inflammation, lipid metabolism, and cellular stress responses.
(b) Oxidative stress reduction, through enhanced antioxidant defenses, including both direct scavenging of reactive oxygen species (ROS) and upregulation of endogenous antioxidant systems such as superoxide dismutase (SOD), catalase, and glutathione peroxidase. In addition, the synergistic action of dietary antioxidants (e.g., vitamins C and E, carotenoids, polyphenols) helps stabilize cellular redox balance, reduce lipid peroxidation, and protect endothelial and mitochondrial function from oxidative damage.
(c) Hormonal regulation, including effects on appetite and satiety hormones such as leptin, ghrelin, peptide YY (PYY), and glucagon-like peptide-1 (GLP-1). The high fiber and healthy fat content of the MedDiet promotes slower gastric emptying and enhanced satiety signaling, while also improving insulin sensitivity, which indirectly influences appetite regulation and energy homeostasis through hypothalamic pathways involved in hunger and reward signaling.

These findings underscore the complexity of diet–health interactions and reinforce the importance of considering dietary patterns as integrated systems rather than isolated components [22,52,78,94,185,200].

## 7. Economic and Social Dimensions: Barriers, Enablers, and System-Level Considerations

**Figure 5:**
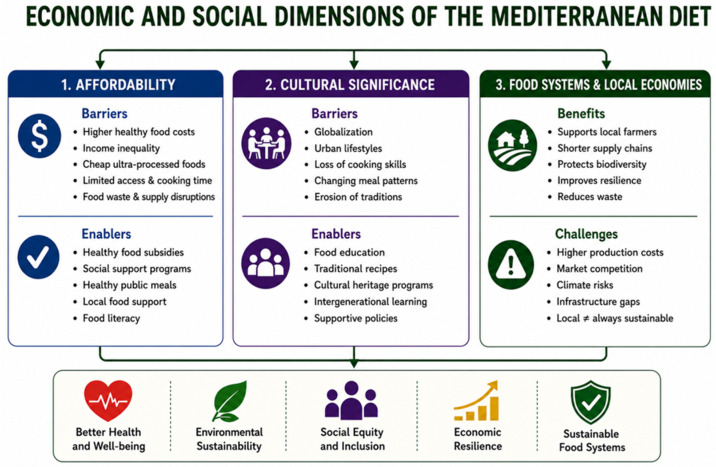
Economic and social dimensions of the Mediterranean diet and the key barriers and enabling factors that influence its adherence.

The MedDiet is increasingly valued for its health, environmental, economic, and social benefits. However, its adoption and long-term sustainability depend on factors such as affordability, cultural relevance, and food-system characteristics. Understanding barriers and facilitators related to economic accessibility, cultural significance, and local food systems is therefore essential for promoting equitable, resilient, and culturally appropriate dietary transitions. In Figure 5, the most crucial economic and social dimensions of the MedDiet and the key barriers and enabling factors that influence its adherence are illustrated.

### 7.1. Affordability

Affordability is a key determinant of dietary behavior and a major challenge in the adoption of sustainable diets. Although the MedDiet is often perceived as expensive because of its emphasis on fresh produce, fish, nuts, and extra virgin olive oil, its affordability is highly context-dependent and should be evaluated in terms of nutrient density, diet quality, and long-term health value rather than food prices alone [207,208,209]. When assessed using nutritional and public-health metrics, the MedDiet frequently compares favorably with Western dietary patterns; however, translating these advantages into real-world dietary behavior remains difficult, particularly for economically disadvantaged populations [207,208,209].

Food choices are often driven by immediate affordability rather than long-term health benefits. Consequently, even if the MedDiet is cost-effective from a societal perspective, higher upfront costs of certain foods may limit adoption among low-income households [207,208]. Modern food environments further reinforce this challenge by increasing the availability of inexpensive, convenient, and heavily marketed ultra-processed foods, creating structural barriers that disproportionately affect lower socioeconomic groups [208,210,211]. Importantly, affordability is influenced not only by food prices but also by factors such as food availability, transportation access, work schedules, housing conditions, cooking facilities, and food literacy [208,209].

Market prices often fail to reflect the true environmental, health, and social costs of food production and consumption. Environmental degradation, biodiversity loss, GHG emissions, and healthcare expenditures associated with unhealthy dietary patterns are largely externalized, making highly processed foods appear economically attractive despite their substantial long-term societal costs [208,211]. In addition, food inflation, supply-chain disruptions, climate-related agricultural impacts, and regional differences in food production contribute to substantial variation in the affordability of Mediterranean dietary patterns across countries and populations [211,212].

Affordability should therefore be viewed as a multidimensional concept encompassing financial, temporal, and social resources. Time constraints, culinary skills, meal planning, and food preparation capacity can be as important as monetary cost, particularly for households balancing multiple jobs or caregiving responsibilities [209,212]. Food waste also influences effective dietary costs; traditional Mediterranean practices such as seasonal cooking, food preservation, and creative reuse of leftovers historically improved resource efficiency, although these practices have become less common in modern food environments [212,213,214].

Policy interventions can help reduce affordability barriers through subsidies for fruits, vegetables, legumes, and other nutrient-dense foods, support for local food systems, strategic taxation policies, and social protection programs targeting vulnerable populations [213,214]. Public procurement initiatives that incorporate Mediterranean-style meals into schools, hospitals, universities, and other institutions may further improve affordability through economies of scale while supporting healthier dietary norms and local agriculture [213,214].

Importantly, the traditional MedDiet—centered on legumes, whole grains, seasonal fruits and vegetables, and modest amounts of animal products—can be economically feasible or even cost-saving when based on locally sourced and minimally processed foods [207,210]. However, commercialized interpretations emphasizing premium, imported, or specialty products may substantially increase costs and reduce accessibility [208,209,210]. Consequently, the affordability of the MedDiet depends largely on how it is defined and implemented.

Overall, affordability should not be considered an inherent characteristic of the MedDiet but rather an outcome of broader socioeconomic, cultural, and policy environments. Improving access to sustainable dietary patterns therefore requires systems-level approaches that address income inequalities, food environments, agricultural policies, food literacy, and social determinants of health, ensuring that healthy and sustainable diets become both accessible and achievable across diverse populations.

### 7.2. Cultural Significance

The MedDiet is more than a nutritional model; it is a culturally embedded lifestyle shaped by traditions, social practices, culinary knowledge, and regional identities [157,215,216]. Its sustainability is partly rooted in the integration of food within broader social, cultural, and environmental contexts, where dietary habits are reinforced through shared norms, communal meals, local food procurement, and intergenerational transmission of knowledge [157,215,216]. These practices contribute not only to dietary adherence but also to social cohesion, psychological well-being, and cultural continuity.

In Mediterranean societies, traditional foods, recipes, seasonal celebrations, local markets, and food customs help preserve cultural heritage, agricultural knowledge, and social values linked to food preparation and consumption [215,216]. Cultural relevance is a major determinant of long-term dietary adherence, making the Mediterranean diet a valuable model for culturally adaptable dietary frameworks that integrate health, sustainability, and local traditions [215,217]. Its core principles—plant-based eating, seasonality, minimally processed foods, and social engagement with food—can be adapted across diverse cultural settings even when Mediterranean foods are not directly available [216,217].

However, the cultural foundations of the MedDiet face growing challenges from globalization, urbanization, demographic change, and evolving lifestyles [218]. Increased consumption of ultra-processed foods, expansion of fast-food and food-delivery services, changing labor patterns, and reduced time for cooking have contributed to declining adherence, particularly among younger generations [218]. These trends have weakened cooking skills, food literacy, communal eating practices, and the intergenerational transmission of traditional dietary knowledge, promoting dietary homogenization and cultural erosion [218].

At the same time, cultural sustainability should not be equated with preserving dietary traditions in a static form. Food cultures continuously evolve in response to migration, technological innovation, economic development, and social change [215,216]. Consequently, sustainable dietary strategies should balance the preservation of beneficial cultural attributes with adaptation to contemporary lifestyles and societal needs [215,216,217,218]. This perspective avoids romanticizing traditional food systems while recognizing the structural factors—such as urban living conditions, time constraints, changing family structures, and economic pressures—that influence dietary behavior [217,218].

Promoting the cultural dimensions of the MedDiet therefore requires more than nutrition education alone. School-based food education, culinary training, intergenerational learning initiatives, community food-heritage programs, and policies supporting local food systems, traditional agriculture, and protected regional products can help maintain cultural continuity and strengthen dietary resilience [217,218]. However, the success of these interventions depends on broader socioeconomic conditions and their ability to address the realities of modern food environments.

Collectively, the cultural significance of the MedDiet lies in its capacity to integrate health, social cohesion, cultural identity, and environmental stewardship within a single dietary framework. Its long-term sustainability will depend on societies’ ability to adapt its core principles to changing social, economic, and cultural conditions while preserving the values that have historically contributed to its resilience and public health benefits.

### 7.3. Food Systems and Local Economies

The MedDiet is closely linked to local food systems, which contribute to economic sustainability, environmental stewardship, cultural heritage, and community resilience [219]. Characterized by shorter supply chains and stronger producer–consumer relationships, these systems help preserve traditional agricultural knowledge, regional food cultures, and agrobiodiversity. However, local food systems should not be considered inherently sustainable, as their environmental, economic, and social performance depends largely on production methods, resource-use efficiency, governance, and market conditions rather than geographic proximity alone [219].

Economically, local food systems can strengthen rural economies by supporting small-scale farmers, increasing local value creation, improving income stability, and generating employment, particularly in Mediterranean regions where traditional farming is maintained by smallholders [220,221]. However, their viability is often constrained by higher production costs, limited market access, labor shortages, aging farming populations, and market volatility, necessitating supportive policies and infrastructure investments [220,221,222].

From an environmental perspective, local food systems may promote seasonal consumption, crop diversification, reduced post-harvest losses, and biodiversity-friendly farming practices [222,223]. However, because agricultural production generally contributes more to food-system emissions than transportation, locally produced foods are not inherently more sustainable than imported alternatives. Therefore, sustainability depends primarily on agroecological practices, efficient resource management, and biodiversity conservation rather than localization alone [222,223].

Local food systems can also enhance food-system resilience by reducing dependence on long supply chains and improving food security during disruptions such as climate change, economic crises, geopolitical instability, and pandemics [224,225]. The COVID-19 pandemic highlighted the vulnerability of global food networks and renewed interest in regional food systems. However, highly localized systems may themselves be susceptible to droughts, pest outbreaks, climate extremes, or regional economic shocks. Consequently, resilience is best achieved through an appropriate balance between local production capacity and broader national and international food networks [224,225].

In addition, local food systems may support circular economy models through composting, reuse of agricultural by-products, artisanal production, and food-waste valorization, thereby improving resource efficiency and retaining economic value within communities [226]. Yet implementation often requires technical expertise, infrastructure, and supportive governance frameworks [226].

Despite their potential benefits, local food systems face increasing pressures from industrial agriculture, market consolidation, demographic change, rural depopulation, and evolving consumer preferences [227]. Their sustainability should therefore not be romanticized, as outcomes depend on labor conditions, production practices, resource management, and market structures rather than geographic proximity alone [225,226,227].

The transferability of the MedDiet beyond Mediterranean regions may also require adaptation to local ecological, cultural, and agricultural conditions. Increasingly, evidence suggests that its global relevance lies less in replicating specific foods than in applying broader principles such as plant-forward eating, seasonality, dietary diversity, minimal processing, and support for local food systems [223,224,225,226].

Overall, local food systems represent an important component of sustainable food-system transformation and reinforce many principles of the MedDiet. Maximizing their contribution requires integrated strategies that combine sustainable agricultural practices, resilient supply chains, supportive policies, and equitable economic structures capable of balancing environmental, social, and economic objectives.

## 8. Barriers to Adoption and Globalization Challenges

The global adoption of the MedDiet is increasingly challenged by structural, socioeconomic, and cultural factors despite its recognized health and sustainability benefits. Dietary transitions, urbanization, and economic inequalities have altered food environments and dietary behaviors, creating barriers to widespread implementation. These challenges highlight the influence of broader food-system dynamics and the need for coordinated, multi-level strategies to preserve and adapt sustainable dietary patterns across diverse populations. In Table 3, the most important barriers to adoption of the MedDiet and the crucial impacts of dietary transitions, urbanization, and economic inequality are summarized.

### 8.1. Dietary Transition

Globalization has profoundly reshaped dietary patterns worldwide, contributing to a widespread nutrition transition characterized by the displacement of traditional dietary patterns—such as the MedDiet—by Westernized diets rich in UPFs [228,229,230,231]. These products, typically industrial formulations high in added sugars, unhealthy fats, sodium, and refined ingredients, have become increasingly dominant in global food systems due to their affordability, convenience, long shelf life, and aggressive marketing strategies [232,233,234]. In addition, their formulation is often designed to maximize palatability and consumption frequency through combinations of refined starches, flavor enhancers, emulsifiers, and additives that may alter satiety signaling and promote overconsumption [230,234].

Evidence indicates a rapid global increase in the production and consumption of UPFs, particularly in middle-income countries undergoing economic development and urbanization [229,231]. This trend has contributed to the erosion of traditional dietary patterns, including reduced consumption of minimally processed plant-based foods central to the MedDiet [100,229]. Even within Mediterranean regions, adherence to traditional dietary practices has declined, especially among younger populations, driven by greater availability of fast foods, digital food delivery services, time constraints, and reduced exposure to home-cooked meals and culinary traditions [235,236].

Importantly, this dietary transition reflects broader structural transformations rather than individual preferences alone. Globalization of supply chains, urbanization, changing labor patterns, agricultural intensification, food industry consolidation, and the expansion of multinational food corporations have created food environments that favor convenience-oriented and highly processed products [237,238]. Agricultural policies and commodity markets have further reinforced this trend by prioritizing crops such as maize, wheat, soy, and sugar, key ingredients in many UPFs, thereby contributing to dietary homogenization and reduced reliance on diverse plant foods [237,238].

From a public health perspective, high UPF consumption has been associated with obesity, type 2 diabetes, cardiovascular disease, and other non-communicable diseases [233,236]. However, the UPF category is highly heterogeneous, and debate remains regarding the relative contributions of food processing, nutritional quality, energy density, and overall dietary patterns to these outcomes [233,236]. Similarly, while UPF-dominated diets are often linked to greater packaging waste, industrialized production systems, and longer supply chains, environmental impacts vary considerably among products and production methods, highlighting the need for whole food-system assessments rather than evaluations based solely on processing level [233,236,238].

The increasing reliance on convenience foods also reflects broader social changes, including urbanization, workforce participation, changing family structures, and limited time for food preparation [230,237]. Consequently, a simple return to traditional dietary patterns may be neither realistic nor feasible. Sustainable dietary strategies must therefore balance preservation of beneficial traditional practices with adaptation to contemporary lifestyles and socioeconomic realities.

Globalization has generated both challenges and opportunities. While it has contributed to dietary homogenization and the decline of local food cultures, it has also improved food availability and facilitated the dissemination of nutritional knowledge [237,238]. Therefore, globalization should not be viewed solely as a threat but as a process requiring careful management to preserve dietary diversity and sustainability.

Overall, dietary transition represents a major barrier to the preservation and broader adoption of the MedDiet. Addressing this challenge will require coordinated actions extending beyond individual behavior change, including reforms in agricultural policy, food marketing, urban food environments, school food programs, and economic incentives that promote healthy and sustainable food choices [233,236,237,238]. Future strategies should focus on adapting Mediterranean dietary principles to modern food environments while preserving their nutritional, environmental, and socio-cultural value.

### 8.2. Urbanization

Urbanization is a major driver of dietary change, influencing food environments, lifestyles, and consumption patterns through shifts in work schedules, family structures, and social interactions [239,240]. Time constraints, increased workforce participation, commuting, and occupational stress have reduced opportunities for meal planning and home cooking, increasing reliance on convenience and ready-to-eat foods [239,240]. Importantly, these changes reflect broader structural transformations involving economic conditions, labor markets, housing patterns, and commercial food systems rather than individual choices alone [239,240].

Urban food environments often promote consumption of ultra-processed, energy-dense foods through their widespread availability, convenience, affordability, and increasing access via fast-food outlets, vending machines, and digital delivery platforms, contributing to dietary patterns that diverge from the Mediterranean diet [228,241,242]. However, urban settings can also support healthier eating through access to diverse foods, farmers’ markets, nutrition education, and innovative food-system initiatives, emphasizing the importance of food-environment design and governance [242].

Urbanization is also associated with declining cooking skills and food literacy, factors linked to higher consumption of ultra-processed foods and reduced ability to plan healthy meals [241,242,243,244]. These trends largely reflect broader socioeconomic changes, including increased workforce participation, evolving family structures, and changing social norms, which have altered the role of cooking in everyday life [241,242,243,244].

Furthermore, urban lifestyles often promote fragmented eating patterns, including frequent snacking, eating outside the home, solitary meals, and reduced communal dining, weakening the social and cultural practices traditionally associated with the MedDiet [218,245]. Increased exposure to food advertising, social media marketing, and digital food promotion further reinforces preferences for highly processed foods, particularly among children, adolescents, and socioeconomically disadvantaged groups [218,245].

In Mediterranean countries, urbanization has contributed to the progressive Westernization of diets and declining adherence to traditional Mediterranean dietary practices, especially among younger populations [100,230]. However, the distinction between urban and rural populations is becoming less pronounced, as globalized food environments increasingly influence both settings [240,241,242].

Importantly, urbanization has also delivered social benefits, including improvements in education, income, healthcare access, food availability, and gender equality [243,244,245]. Consequently, sustainable dietary strategies should not seek a return to past lifestyles but rather adapt the core principles of the MedDiet to contemporary urban realities. At the same time, urbanization often exacerbates socioeconomic inequalities, creating disparities in food affordability, availability, and quality, particularly in lower-income neighborhoods where access to healthy foods is limited and exposure to unhealthy food outlets is greater [241,242].

Addressing these challenges requires systems-level interventions extending beyond individual behavior change. Urban planning, food-system governance, public procurement, transportation policies, and economic incentives can help create healthier food environments. Initiatives such as urban agriculture, farmers’ markets, healthy retail programs, workplace nutrition policies, and school-based food education may support healthier and more sustainable dietary behaviors [100,230].

Conclusively, urbanization represents both a challenge and an opportunity for the future of the MedDiet. While contemporary urban food environments have contributed to declining adherence to traditional dietary patterns, cities also provide important opportunities for innovation and food-system transformation. The key challenge is not to resist urbanization but to redesign urban food environments in ways that support the health, cultural, and sustainability principles of the Mediterranean dietary model.

### 8.3. Economic Inequality

Economic inequality is a major barrier to the adoption and sustainability of the MedDiet, influencing both food access and dietary choices. Although the traditional MedDiet can be affordable when based on legumes, whole grains, seasonal produce, and modest amounts of animal products, healthy minimally processed foods are often more expensive and less accessible than ultra-processed alternatives in contemporary food environments [207,208,246]. Affordability is also shaped by factors such as time availability, transportation, cooking facilities, food storage capacity, and overall household resources, which disproportionately affect disadvantaged populations [207,208,246].

Lower-income populations often face barriers to Mediterranean diet adherence because healthy foods such as fruits, vegetables, fish, nuts, and olive oil are relatively expensive, whereas ultra-processed foods are inexpensive, convenient, widely available, and heavily marketed [228,229,247]. Price volatility of fresh foods can further exacerbate these challenges [228,229,247]. Moreover, dietary inequalities are driven not only by food costs but also by economic insecurity, which prioritizes short-term food sufficiency, and by market structures that fail to account for the environmental, social, and healthcare costs of unhealthy diets [207,208,246].

Food environments further reinforce disparities. Food deserts, limited transportation, unequal retail distribution, and the concentration of fast-food outlets in deprived neighborhoods reduce access to healthy foods while increasing exposure to unhealthy options [228,229,240,243,248]. Educational inequalities also contribute through differences in nutrition knowledge, food literacy, and cooking skills, although information alone is insufficient to overcome broader economic and environmental barriers [240,245,249,250].

These factors contribute to a social gradient in diet quality; whereby healthier dietary patterns are more common among higher-income and better-educated populations [248,249,250]. As a result, sustainable dietary recommendations risk widening health inequalities if accessibility and affordability barriers are not addressed. Similar challenges exist globally, as differences in agricultural systems, food prices, trade structures, and cultural contexts may limit the direct transferability of the MedDiet to non-Mediterranean regions [212,241,251]. Consequently, emphasis should be placed on adapting Mediterranean principles rather than replicating specific foods [212,241,251].

Economic barriers to healthy eating are closely linked to broader social determinants of health, including income insecurity, precarious employment, housing instability, social exclusion, and unequal access to healthcare [212,241,251]. Addressing these challenges requires integrated policies such as healthy food subsidies, social protection measures, agricultural reforms, investments in local food systems, equitable food-pricing strategies, and healthy meal programs in schools, workplaces, universities, and healthcare settings [231,251].

Finally, economic inequality is not merely a barrier to MedDiet adoption but a fundamental determinant of whether sustainable food systems can achieve both public health and social justice goals. Ensuring equitable access to sustainable diets requires systems-level interventions that address affordability, accessibility, and the broader socioeconomic conditions shaping dietary behavior.

## 9. Policy Implications and Future Directions

**Figure 6:**
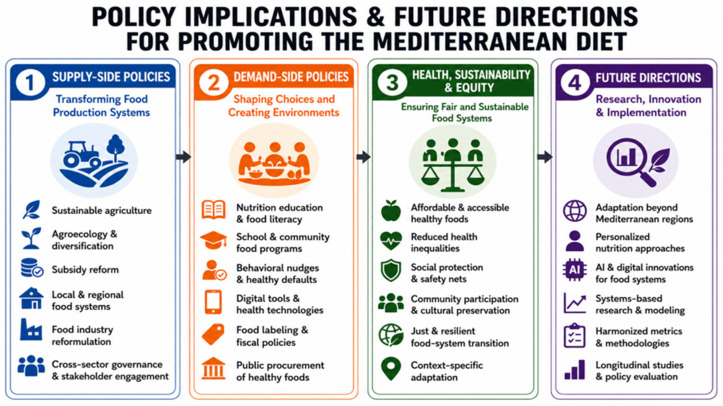
Policy implications and future directions promoting the Mediterranean diet for health sustainability and equity.

The promotion and large-scale adoption of the MedDiet as a sustainable dietary model requires comprehensive, multisectoral policy frameworks that address both supply-side and demand-side determinants of dietary behavior. Given the complexity of modern food systems, isolated interventions are unlikely to achieve meaningful or sustained impact. Instead, coordinated strategies spanning agriculture, public health, education, economics, and environmental policy are essential [28,194,252]. In Figure 6, the policy implications and future directions promoting the MedDiet for health sustainability and equity are illustrated.

### 9.1. Supply-Side Interventions: Transforming Food Production Systems

Agricultural and food-system policies play a central role in determining the availability, affordability, and sustainability of foods. Promoting dietary patterns aligned with the MedDiet requires a transition toward more diverse, resilient, and sustainable production systems. However, this transformation extends beyond increasing the production of Mediterranean foods, as current food systems are shaped by global markets, trade policies, and economic incentives that often prioritize productivity and competitiveness over health and sustainability goals [253,254].

A major obstacle is fragmented governance across agriculture, environment, trade, public health, and economic sectors, which frequently results in conflicting policies. For example, public health recommendations promoting fruits, vegetables, and legumes may coexist with agricultural incentives favoring commodity crops and livestock production [253,254]. Strengthening cross-sectoral governance can improve policy coherence, although competing economic interests, stakeholder priorities, and political pressures often complicate implementation [253,254].

Research, innovation, and sustainable farming practices—including precision agriculture, climate-resilient crops, digital technologies, agroecology, regenerative agriculture, and organic farming—can enhance resource efficiency, biodiversity, soil health, climate resilience, and the production of MedDiet foods [253,254]. However, technological advances alone cannot overcome structural challenges such as market concentration, social inequalities, limited land access, and unsustainable consumption patterns, while agroecological approaches may face scalability and productivity trade-offs [253,254].

Agricultural subsidy reform is another key priority. In many regions, subsidies continue to favor livestock production and commodity crops, distorting food prices and limiting the competitiveness of nutrient-dense plant foods [152,255]. Redirecting support toward diversified cropping systems, sustainable agriculture, and smallholder farming could facilitate healthier and more sustainable diets. However, such reforms are politically sensitive and must balance environmental objectives with economic stability and social equity [152,255].

Strengthening local and regional food systems may further enhance resilience, support rural economies, and improve access to fresh foods through shorter supply chains, farmers’ markets, cooperatives, and regional food networks [256]. However, local production should not automatically be considered more sustainable, as environmental outcomes depend on production practices, resource efficiency, and overall supply-chain performance rather than proximity alone [256].

Food industry engagement is also important. Product reformulation, improved food labeling, and the development of healthier, minimally processed, and plant-based foods can help align food environments with MedDiet principles [257]. However, voluntary industry actions have often produced limited progress, and stronger regulatory measures may be required to ensure meaningful improvements in public health and sustainability outcomes [257].

Notably, supply-side interventions alone are insufficient to drive large-scale dietary change. Greater availability of healthy foods does not necessarily translate into increased consumption if economic, cultural, and behavioral barriers persist [256,257]. Therefore, production-side reforms should be integrated with demand-side measures, including nutrition education, food literacy, public procurement programs, and policies that address affordability and social inequalities.

Finally, international cooperation can support sustainable dietary transitions through the exchange of knowledge, best practices, and policy innovations. However, implementation must remain context-specific, reflecting differences in agricultural systems, cultural traditions, governance structures, and socioeconomic conditions. Overall, transforming food systems to support Mediterranean dietary principles is not merely a technical challenge but a complex political, economic, and social process requiring integrated governance capable of balancing productivity, sustainability, public health, and equity objectives.

### 9.2. Demand-Side Strategies: Shaping Consumer Behavior

Demand-side policies are essential for promoting adherence to the MedDiet by addressing not only nutrition knowledge but also the behavioral, social, economic, commercial, and environmental factors that shape food choices [258]. Evidence suggests that educational interventions alone often produce modest and short-lived effects when structural barriers remain in place. Consequently, sustainable dietary transitions require systems-based approaches that make healthy choices accessible, affordable, convenient, and socially supported [258].

Food preferences are strongly influenced by social norms, cultural values, family dynamics, peer networks, food marketing, and socioeconomic conditions [258,259]. Community-based initiatives and targeted interventions can help normalize healthier eating behaviors and reduce health inequalities, but their effectiveness is limited if food environments continue to favor unhealthy options [258,259].

Nutrition education and food literacy remain important foundations for dietary change. Programs emphasizing practical skills such as meal planning, cooking, budgeting, food selection, and nutrition-label interpretation can support healthier and more sustainable food choices, particularly when introduced early through school curricula and experiential activities such as school gardens, cooking workshops, and farm-to-school programs [258,259]. However, knowledge alone is often insufficient to overcome barriers related to affordability, food access, time constraints, and commercial influences [258,259].

School-based interventions, community programs, and mass-media campaigns have demonstrated positive effects on dietary behavior, especially when culturally tailored and supported by enabling food environments [260,261]. Nevertheless, long-term sustainability is often limited, and outcomes vary according to socioeconomic conditions and local food environments [260,261].

Behavioral strategies such as nudging—including improved placement and visibility of healthy foods, portion-size standardization, and healthier default options—can facilitate healthier choices while preserving individual autonomy [262,263]. Digital health tools, including mobile applications and personalized nutrition platforms, may further support adherence to the MedDiet, although their effectiveness varies and unequal access may exacerbate health disparities [262,263].

Regulatory and fiscal measures are increasingly recognized as powerful tools for shaping food environments. Front-of-pack nutrition labeling, taxes on ultra-processed foods and sugar-sweetened beverages, and subsidies for healthier alternatives can influence purchasing behavior and dietary quality [264,265]. However, such policies must be carefully designed to avoid disproportionately burdening lower-income populations and to ensure equitable health benefits [265].

Restrictions on the marketing of unhealthy foods, particularly to children, are also important. The rapid expansion of digital marketing and social media advertising has increased exposure to unhealthy food promotion, especially among younger populations, highlighting the need for updated regulatory frameworks [263,264,265].

Public procurement policies offer another effective mechanism for promoting sustainable diets. Incorporating MedDiet principles into meals provided in schools, hospitals, universities, and other public institutions can improve diet quality, reduce inequalities, and stimulate demand for sustainable food production [263,264,265]. Successful implementation, however, requires adequate resources, infrastructure, and political commitment.

Critically, the effectiveness of demand-side interventions is constrained by the broader commercial determinants of health. Food corporations influence dietary choices through product formulation, pricing, retail placement, branding, and marketing practices [260,261,262]. Therefore, meaningful dietary transformation requires not only consumer education but also policies that address the structural and commercial drivers of unhealthy food environments.

Eventually, promoting adherence to the MedDiet requires an integrated approach combining nutrition education, behavioral strategies, fiscal and regulatory policies, public procurement, and supportive food environments. Long-term success depends less on individual responsibility alone and more on creating food systems in which healthy and sustainable choices become the easiest and most accessible options.

### 9.3. Integrating Health, Sustainability, and Equity

Ensuring equitable adoption of the MedDiet requires addressing disparities in access to healthy and sustainable foods. Vulnerable populations often face overlapping barriers, including limited income, poor food environments, unstable employment, time constraints, low food literacy, and restricted access to nutrition education, which contribute to persistent dietary inequalities and increased risk of diet-related diseases. A life-course perspective is therefore needed, recognizing the long-term influence of early socioeconomic conditions, education, and food environments on dietary behavior and health outcomes.

Notably, dietary inequalities are rooted not only in individual choices but also in broader social, economic, and political structures. Poverty, income inequality, social exclusion, labor insecurity, and unequal access to public services can limit the ability of individuals and communities to adopt healthy dietary patterns. Consequently, sustainable dietary policies must address these structural determinants alongside behavior-change interventions.

Gender and household dynamics also influence dietary practices. Women often carry a disproportionate share of food-related responsibilities, meaning that recommendations emphasizing home cooking may inadvertently increase unpaid labor burdens if gender inequalities are not considered. Similarly, community food networks, informal food economies, and culturally tailored interventions can improve access, resilience, and acceptability among diverse populations.

Integrating nutrition and sustainability goals into broader policies—including urban planning, transportation, housing, education, and social protection—can create environments that support healthier dietary choices. Measures such as urban agriculture, community-supported agriculture, mobile markets, food hubs, and improved transportation access may enhance food availability and affordability, although effectiveness depends on local socioeconomic and cultural contexts [266,267].

Social protection programs, including food vouchers, targeted subsidies, and income-support measures, can improve dietary quality among vulnerable groups. However, financial assistance alone is often insufficient if broader structural inequalities remain unaddressed. Therefore, such interventions should be embedded within wider poverty-reduction and equity strategies.

Effective implementation also requires collaboration among governments, researchers, civil society, and private stakeholders. While cross-sector partnerships can improve coordination and resource mobilization, transparency and accountability are essential to manage conflicts of interest and ensure that public health objectives are not compromised by commercial priorities.

Robust monitoring and evaluation systems are needed to assess impacts on dietary behavior, health outcomes, and social inequalities. Greater emphasis should be placed on long-term structural changes and differential effects across socioeconomic groups to ensure that interventions reduce rather than exacerbate disparities. Investments in food infrastructure, distribution systems, and community participation can further strengthen equitable access to healthy foods.

Equity considerations must also extend globally. Sustainable dietary strategies should support culturally appropriate adaptations rather than imposing a single dietary model, particularly in low- and middle-income countries where priorities related to food security, undernutrition, agricultural development, and economic growth may differ substantially [266,267]. Increasingly, researchers advocate adapting the principles of the MedDiet to local food systems, cultural traditions, and agricultural realities.

At the same time, sustainability policies may generate unintended social consequences. Measures such as taxes on environmentally intensive foods or restrictions on specific products can disproportionately affect low-income consumers and producers unless accompanied by compensatory measures. Consequently, the concept of a just transition has become central to food-system transformation, emphasizing the need to balance environmental goals with social equity and economic inclusion.

International cooperation, capacity building, and policy coherence across trade, agriculture, nutrition, and sustainability sectors can further support equitable dietary transitions. However, interventions should promote reciprocal learning and recognize the value of local knowledge, indigenous food systems, and region-specific sustainability practices.

Ultimately, achieving sustainable diets requires more than improving food choices; it requires transforming the social, economic, and political conditions that shape food systems. Ensuring fair labor conditions, equitable value distribution, social protection, and universal access to healthy foods is essential if sustainability objectives are to advance both public health and social justice.

### 9.4. Future Research Directions

#### 9.4.1. Adaptability to Non-Mediterranean Contexts

Despite substantial evidence supporting the MedDiet, several research gaps remain that warrant further investigation. One key area is the adaptability of the MedDiet to non-Mediterranean contexts, where differences in climate, food availability, agricultural systems, food affordability, and cultural preferences may necessitate context-specific modifications [157,268]. Research should focus on identifying core principles—such as plant predominance, seasonal eating, culinary simplicity, and reliance on minimally processed foods—that can be translated into locally appropriate dietary patterns while maintaining both nutritional adequacy and environmental sustainability.

#### 9.4.2. Comparative Research and Methodological Harmonization

More comparative studies are needed to evaluate Mediterranean dietary patterns across diverse socioeconomic and ecological contexts, particularly in low- and middle-income countries facing the double burden of malnutrition. Harmonized methods for assessing Mediterranean diet adherence, along with standardized indicators of nutritional quality and environmental sustainability, would improve cross-study comparability. Incorporating context-specific factors such as cultural acceptability, food accessibility, and economic constraints, and strengthening international research collaborations and data-sharing initiatives, could further enhance the relevance and global applicability of findings.

#### 9.4.3. Policy and Food System Contexts

Furthermore, greater attention should be given to the role of policy environments and food system structures in shaping the feasibility of adapting the MedDiet across regions. Expanding the use of standardized dietary assessment tools and sustainability metrics can also improve the robustness and consistency of future research. In addition, incorporating qualitative research approaches may help capture cultural and behavioral dimensions that are often overlooked in quantitative analyses. Finally, more region-specific case studies can provide practical insights into successful adaptation strategies and implementation pathways.

#### 9.4.4. Personalized Nutrition and Precision Public Health

Another important avenue is the integration of the MedDiet with emerging fields such as personalized nutrition and precision public health. Advances in genomics, metabolomics, proteomics, and microbiome research offer opportunities to tailor dietary recommendations to individual biological characteristics, potentially enhancing effectiveness and adherence [269]. However, further evidence is needed to determine whether personalized approaches can be implemented at population scale without exacerbating existing health inequalities, particularly given disparities in access to advanced healthcare technologies, diagnostic tools, and digital infrastructure. Moreover, ethical considerations regarding data ownership, algorithm transparency, and commercialization of health data require further attention.

#### 9.4.5. Digital Technologies and Innovation

Digital technologies also hold promise for scaling interventions. Mobile applications, wearable devices, and online platforms can support dietary monitoring, behavior change, and education, making the MedDiet more accessible to diverse populations [270]. The integration of artificial intelligence and data-driven feedback systems may further enhance personalization and engagement, enabling real-time dietary feedback and adaptive interventions. However, issues related to data privacy, cybersecurity, digital literacy, and equitable access must be carefully addressed to avoid widening the digital divide, particularly among older adults and socioeconomically disadvantaged groups.

#### 9.4.6. Systems-Based and Longitudinal Research Approaches

Future research should prioritize longitudinal and systems-based approaches that capture the complex interactions between diet, health, environment, and society. Such approaches should incorporate life-course perspectives, food system modeling, and real-world intervention studies to better understand causal pathways and long-term outcomes, including intergenerational effects of dietary patterns. Interdisciplinary collaboration between nutrition science, environmental research, behavioral science, economics, and policy studies will be essential to generate robust evidence and inform effective, scalable policy decisions capable of supporting global dietary transitions toward sustainability.

### 9.5. Toward a Systems-Based Transformation

Eventually, the promotion of the MedDiet requires a paradigm shift from individual-focused interventions to systems-based transformation. This involves aligning policies across sectors, engaging stakeholders throughout the food system, and addressing the structural drivers of dietary behavior, including food pricing mechanisms, agricultural subsidies, urban food environments, and global trade dynamics. Such an approach also requires recognizing that dietary choices are shaped by complex interactions between biological, social, economic, and environmental determinants rather than individual willpower alone. It also implies shifting performance metrics from short-term behavioral outcomes to long-term system-level impacts, including sustainability, resilience, and equity. In this regard, adopting a “food systems lens” can help identify leverage points for intervention and facilitate more holistic and coordinated strategies.

It further necessitates the integration of governance frameworks that can coordinate actions across local, national, and global levels, ensuring policy coherence and long-term sustainability. In this context, fostering partnerships between governments, academia, civil society, and the private sector is essential to mobilize resources and drive systemic change. Additionally, empowering communities through participatory approaches can enhance the relevance, acceptance, and effectiveness of dietary interventions. Strengthening institutional capacity and policy alignment across sectors can also reduce fragmentation and improve implementation efficiency. Furthermore, incorporating indigenous knowledge and traditional practices can enrich policy design and support culturally grounded food system solutions.

The MedDiet provides a unique opportunity to bridge the gap between public health, environmental sustainability, and cultural heritage, offering a model for integrated policy action. Its multidimensional benefits—including reduced risk of non-communicable diseases, lower environmental footprints, and preservation of traditional food cultures—position it as a cornerstone for sustainable food system transformation. However, realizing its full potential will depend on sustained political commitment, cross-sector collaboration, and the ability to adapt its principles to diverse and rapidly changing global contexts. In addition, effective implementation will require monitoring and evaluation frameworks to track health, environmental, and socioeconomic outcomes, ensuring that policies remain evidence-based and responsive to emerging global challenges such as climate change, food insecurity, and rising dietary inequalities. Establishing standardized indicators and benchmarks can further enhance accountability and facilitate international comparisons of progress. Moreover, integrating scenario modeling and foresight analysis can help policymakers anticipate future challenges and design more resilient strategies.

Strengthening capacity-building initiatives and investing in workforce development across sectors can further support the transition toward sustainable dietary systems. Moreover, integrating sustainability considerations into national dietary guidelines and public procurement policies can institutionalize the MedDiet’s principles within formal governance structures. Finally, leveraging innovation and technological advancements can enhance scalability and accelerate progress toward resilient and equitable food systems. Expanding access to data-driven decision-making tools can also support more targeted and efficient policy interventions. In addition, fostering innovation ecosystems that connect researchers, entrepreneurs, and policymakers can accelerate the development and dissemination of sustainable food solutions.

## 10. Strengths and Limitations

**Figure 7:**
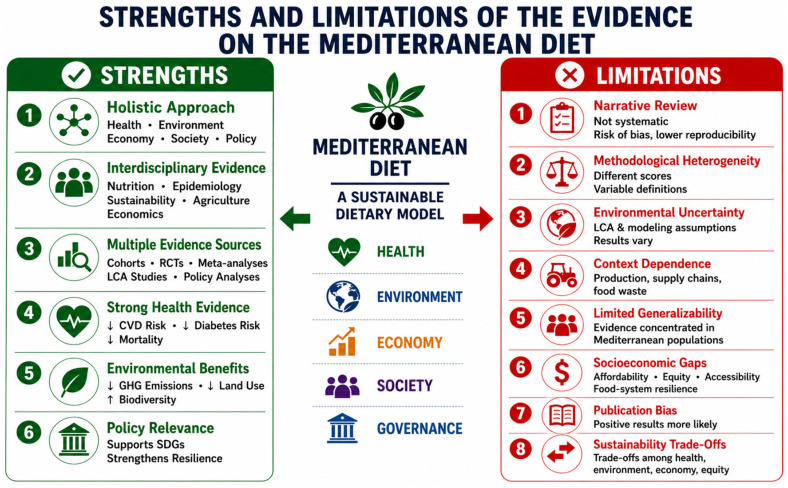
Strengths and limitations of the evidence on the Mediterranean diet. ↓ means “decrease” and ↑ means “increase”.

This state-of-the-art review provides a comprehensive and interdisciplinary synthesis of the MedDiet as a model of a sustainable dietary pattern, integrating evidence from nutrition science, epidemiology, environmental sustainability, public health policy, agricultural systems, and socio-economic research [271]. By combining findings across multiple domains, the review moves beyond reductionist nutritional perspectives and adopts a systems-based approach that reflects the complexity of contemporary food systems. However, both the strengths and limitations of the available evidence must be carefully considered when interpreting the findings (Figure 7).

A major strength of this review lies in its holistic conceptual framework, which simultaneously examines the nutritional, environmental, economic, and socio-cultural dimensions of sustainability. This multidimensional perspective is increasingly recognized as essential because dietary patterns influence not only health outcomes but also natural resource use, ecosystem integrity, social equity, and cultural resilience. By integrating evidence from diverse disciplines, the review provides a broader understanding of the interactions between dietary behaviors and the structural determinants of food systems, including agricultural production, food environments, governance structures, and socioeconomic conditions.

Another important strength is the inclusion of evidence from multiple levels of scientific inquiry, including prospective cohort studies, RCTs, systematic reviews, meta-analyses, LCA studies, food-system modeling analyses, and policy evaluations. This triangulation of evidence enhances confidence in several core conclusions, particularly regarding the associations between Mediterranean dietary patterns and cardiometabolic health outcomes, as well as the generally lower environmental impacts of plant-forward dietary patterns compared with Western diets.

Furthermore, the review emphasizes the translational relevance of the MedDiet by situating it within contemporary global challenges, including climate change, biodiversity loss, food insecurity, and the growing burden of non-communicable diseases. This policy-oriented perspective highlights the potential contribution of Mediterranean dietary principles to ongoing efforts aimed at achieving the Sustainable Development Goals (SDGs), strengthening food-system resilience, and promoting planetary health.

Nevertheless, several important limitations should be acknowledged. First, despite efforts to apply a transparent and structured search strategy, this review remains a narrative state-of-the-art synthesis rather than a formal systematic review or meta-analysis. Consequently, study selection, interpretation, and thematic emphasis may be influenced by authorial judgment and selection bias [272]. Although narrative approaches are particularly valuable for integrating heterogeneous evidence across disciplines, they inherently provide lower methodological reproducibility than systematic evidence syntheses.

Second, the evidence base itself is characterized by substantial methodological heterogeneity. There is considerable variation in the operationalization and measurement of MedDiet adherence across studies. Multiple scoring systems—including the MedDiet Score, alternate MedDiet indices, MedDiet Adherence Screener (MEDAS), and region-specific adaptations—use different food group definitions, weighting systems, and adherence thresholds [273]. This variability complicates direct comparisons across studies and may partly explain inconsistencies in reported associations between dietary adherence and health outcomes. Greater harmonization of dietary assessment methodologies remains a priority for future research.

Third, the strength of evidence differs substantially across sustainability dimensions. The health benefits of the MedDiet are supported by a relatively large body of epidemiological and clinical evidence. In contrast, evidence regarding environmental sustainability is derived predominantly from modeling studies and LCA, which are highly dependent on assumptions regarding production systems, system boundaries, allocation methods, and geographic context. Consequently, estimates of greenhouse gas emissions, water footprints, land use, and biodiversity impacts may vary considerably across studies, limiting direct comparability and introducing uncertainty into sustainability assessments.

A related limitation is that environmental sustainability outcomes are often presented as intrinsic properties of the MedDiet, whereas they are strongly influenced by agricultural practices, supply-chain characteristics, food waste levels, and local ecological conditions. For example, intensive irrigated agriculture, greenhouse production, imported foods, or unsustainable fisheries may substantially alter the environmental performance of Mediterranean dietary patterns. Therefore, sustainability outcomes cannot be attributed solely to dietary composition and should instead be understood as emerging from interactions between dietary choices and food-production systems.

Fourth, much of the available evidence originates from Mediterranean populations, particularly cohorts from Southern Europe [274]. While these populations provide valuable insights into traditional dietary practices, the geographic concentration of evidence may limit the external validity and generalizability of findings. Cultural norms, food availability, agricultural systems, culinary traditions, and socioeconomic conditions vary substantially across regions, raising important questions regarding the transferability of the MedDiet to non-Mediterranean settings. Future research should prioritize culturally adapted models of sustainable diets and evaluate their effectiveness in diverse populations.

Fifth, several socio-economic dimensions of sustainability remain underexplored. Although increasing attention has been directed toward affordability, accessibility, equity, and food-system resilience, the evidence base remains less developed than that for health outcomes. Furthermore, economic analyses often fail to fully account for indirect costs, social inequalities, labor conditions, or distributional impacts across different population groups. As a result, important questions remain regarding the feasibility and equity implications of large-scale dietary transitions.

Another limitation concerns the potential for publication bias and positive framing within the sustainable diet literature. Studies demonstrating favorable health or environmental outcomes are more likely to be published than studies reporting null or contradictory findings. Moreover, the MedDiet is frequently presented as a benchmark model of sustainability, which may inadvertently contribute to confirmation bias and underrepresentation of evidence highlighting limitations, trade-offs, or implementation challenges. Greater emphasis on comparative analyses and critical evaluation of competing dietary models may strengthen future research in this field.

Additionally, the concept of sustainability itself remains inherently complex and multidimensional. Trade-offs frequently exist among environmental, economic, social, and health objectives, yet these trade-offs are often insufficiently addressed in the literature. For example, foods associated with low greenhouse gas emissions may exhibit high water footprints, while environmentally beneficial policies may generate affordability challenges for vulnerable populations. Consequently, sustainability assessments should be interpreted with caution and avoid assuming that improvements in one dimension necessarily translate into improvements across all dimensions.

Finally, the field of sustainable diets is evolving rapidly. Emerging research in precision nutrition, microbiome science, food-system resilience, climate adaptation, alternative proteins, and integrated planetary health modeling continues to reshape understanding of the relationships between diet, health, and sustainability. As a result, some conclusions presented in this review may require revision as new evidence becomes available.

In summary, this review provides a broad and integrative synthesis of the MedDiet as a sustainable dietary model and highlights its considerable potential to contribute to human and planetary health. However, the conclusions should be interpreted in light of methodological limitations, evidence heterogeneity, context dependency, and unresolved trade-offs within the sustainability literature. These limitations also identify important priorities for future research, including standardized methodologies, cross-cultural validation, longitudinal systems-based analyses, stronger evaluation of equity outcomes, and more comprehensive assessment of the interactions between dietary patterns and food-system sustainability. Such efforts will be essential for strengthening the evidence base and informing more effective and equitable dietary transitions in the future.

## 11. Conclusions

The MedDiet is among the most extensively studied and empirically supported models of a sustainable dietary pattern, integrating health, environmental, economic, and socio-cultural dimensions within a systems-based framework. Beyond its nutritional composition, the MedDiet reflects broader interactions among dietary behaviors, food systems, cultural traditions, and ecological processes, making it a prominent reference model for sustainable food-system transformation.

A substantial body of evidence links adherence to the MedDiet with reduced risk of major NCDs, improved metabolic health, and increased longevity. Its emphasis on plant-based foods, dietary diversity, seasonality, and moderate consumption of animal-source products is also generally associated with lower environmental impacts than Western dietary patterns. These characteristics position the MedDiet as a valuable framework for addressing interconnected challenges related to climate change, resource depletion, biodiversity loss, and diet-related disease.

However, the sustainability benefits of the MedDiet are context-dependent rather than intrinsic. Health and environmental outcomes are influenced by factors such as production practices, supply chains, resource availability, socioeconomic conditions, food accessibility, and long-term adherence. Consequently, sustainability should be evaluated within the broader food-system context rather than solely through dietary composition.

The MedDiet also faces important challenges. Globalization, urbanization, changing lifestyles, and the expansion of UPFs have contributed to declining adherence, even within Mediterranean countries. Furthermore, direct transfer of the traditional Mediterranean dietary pattern to other regions may not always be feasible due to differences in culture, food availability, agricultural systems, and economic conditions. Therefore, sustainable dietary transitions are likely to be most effective when Mediterranean principles—such as plant-forward eating, dietary diversity, seasonality, moderation, and social connectedness—are adapted to local contexts.

Widespread adoption additionally requires addressing structural barriers, including economic inequality, food insecurity, unsupportive food environments, and unequal access to healthy foods. Educational and behavioral interventions alone are unlikely to achieve lasting change without complementary reforms in agriculture, food policy, trade, urban planning, and social protection systems. Effective policy frameworks must balance environmental, economic, social, and public health objectives while ensuring affordability, equity, and cultural relevance.

Overall, the MedDiet should be viewed not as a universal solution but as a flexible reference model that demonstrates how dietary practices can simultaneously support human health, environmental sustainability, and cultural continuity. Its enduring value lies less in specific foods than in the broader principles it embodies. Future sustainable dietary strategies should build upon these principles while embracing contextual adaptation and addressing the structural determinants that shape food choices. Such an approach will be essential for developing resilient, equitable, and sustainable food systems capable of supporting both human and planetary health.

## Figures and Tables

**Table 1:** Exploring the environmental sustainability dimensions of the Mediterranean Diet: Potential benefits, key mechanisms, major limitations/trade-offs, and critical considerations.

Sustainability Dimension	Potential Benefits	Key Mechanisms	Major Limitations/Trade-Offs	Critical Considerations	Representative References
**GHG Emissions**	Lower food-related GHG emissions than Western diets; estimated reductions of ~30–50%	Reduced red meat consumption; increased plant-food intake; lower methane emissions from ruminants.	Environmental impacts vary according to production systems, transport, processing, and fisheries management.	Benefits are maximized when combined with sustainable agriculture, renewable energy, and food-system transformation.	[127,128,129,130,164,165,166,167,168]
**Land Use and Agricultural Efficiency**	Lower agricultural land demand; reduced pressure on forests and natural ecosystems.	Greater reliance on legumes, cereals, fruits, and vegetables; improved feed-to-food conversion efficiency.	Intensive crop production may still contribute to soil degradation, habitat loss, and biodiversity decline.	Requires agroecological management, regenerative agriculture, and biodiversity-friendly farming.	[169,170,171,172,173,174,175]
**Water Use and Resource Efficiency**	Generally lower water footprint than livestock-rich Western diets.	Reduced dependence on feed production and livestock systems.	Some Mediterranean staples (olive oil, nuts, fruits, vegetables) may require substantial irrigation.	Water sustainability depends on local scarcity, irrigation efficiency, climate conditions, and governance.	[176,177,178,179,180,181]
**Biodiversity Conservation**	Supports agricultural diversity, traditional varieties, and multifunctional agroecosystems.	Demand for diverse crops; maintenance of local landraces and agroforestry systems.	Biodiversity benefits are not automatic; intensive monocultures may negate advantages.	Requires conservation policies, protection of genetic resources, and agroecological farming.	[182,183,184,185,186,187,188,189]
**Food Waste and Resource Conservation**	Reducing food waste lowers pressures on land, water, biodiversity, and climate.	Reduced demand for food production and resource use.	Fresh and perishable foods may increase waste if poorly managed.	Waste prevention should be integrated into sustainability strategies.	[180,181]
**Overall Food-System Sustainability**	Aligns human health and environmental sustainability objectives.	Integration of plant-based eating patterns, traditional food systems, and resource-efficient production.	Outcomes remain highly context-dependent and influenced by policy and consumer behavior.	Requires a systems-based approach encompassing climate, land, water, biodiversity, and governance.	[122,123,125,127,128,129,130,167,168,188,189]

**Table 2:** Key molecular mechanisms underlying the health benefits of the Mediterranean Diet.

Mechanism	Main Molecular Targets	Major Biological Effects	Health Outcome	Representative References
**Anti-inflammatory**	NF-κB, IL-6, TNF-α, SCFAs	↓ Inflammation, ↑ inflammation resolution	Reduced chronic disease risk	[200,201]
**Cardiovascular protection**	HDL, LDL, NO, Eicosanoids	↑ HDL, ↓ LDL oxidation, ↑ endothelial function	Reduced CVD risk and atherosclerosis	[202,203]
**Glycemic regulation**	AMPK, GLP-1, Insulin receptor	↑ Insulin sensitivity, ↓ glucose absorption	Reduced T2DM and metabolic syndrome risk	[196,197]
**Gut microbiota modulation**	SCFAs, GPR41/43, gut microbiota	↑ Beneficial bacteria, ↑ gut barrier integrity	Improved metabolic and immune health	[204,205,206]
**Cancer prevention**	ROS, NF-κB, apoptosis, IGF-1	↓ DNA damage, ↑ apoptosis, ↓ angiogenesis	Reduced cancer risk and progression	[57,61,73,198,199]
**Epigenetic regulation**	DNMTs, HDACs, microRNAs	Modulation of gene expression	Improved metabolic and inflammatory regulation	[200,201,202,203,204,205]
**Oxidative stress reduction**	ROS, SOD, catalase, GPx	↑ Antioxidant defenses, ↓ oxidative damage	Protection against chronic diseases	[200,201,202,203,204,205]
**Hormonal regulation**	Leptin, ghrelin, PYY, GLP-1	↑ Satiety, improved energy balance	Better weight control and metabolic health	[196,197,200,201,202,203,204,205]

↓ means “decrease” and ↑ means “increase”.

**Table 3:** Major barriers to the adoption and globalization of the Mediterranean diet.

Barrier	Key Drivers	Impact on MedDiet Adherence	Potential Solutions	Representative References
**Dietary Transition**	Expansion of UPFs, globalization, food industry consolidation	Reduced intake of traditional plant foods; declining adherence, especially among youth	Food marketing regulation, healthier food environments, school nutrition programs	[228,229,230,231,232,233,234,235,236,237,238]
**Ultra-Processed Food Dominance**	Low cost, convenience, aggressive marketing	Replacement of minimally processed foods; poorer diet quality	UPF reduction strategies, food labeling, consumer education	[229,230,231,232,233,234,235,236]
**Agricultural & Food-System Transformation**	Commodity crop production (maize, wheat, soy, sugar), global supply chains	Dietary homogenization and loss of dietary diversity	Diversified agriculture, local food systems, policy reform	[237,238]
**Urbanization**	Time constraints, commuting, changing family structures	Less home cooking and more convenience-food consumption	Urban food policies, workplace and school interventions	[239,240,241,242,243,244,245]
**Urban Food Environment**	Fast-food outlets, delivery platforms, unhealthy food marketing	Increased consumption of energy-dense, nutrient-poor foods	Healthy retail initiatives, improved food access	[228,241,242,245]
**Declining Food Literacy**	Reduced cooking skills and culinary knowledge	Greater dependence on processed foods	Culinary education, food-literacy programs	[241,242,243,244]
**Economic Inequality**	Income insecurity, higher perceived cost of healthy foods	Reduced access to fruits, vegetables, fish, nuts, and olive oil	Subsidies, social protection, affordability policies	[207,208,246,247]
**Food Access Inequalities**	Food deserts, poor transport, unequal retail distribution	Limited availability of healthy foods	Investments in food infrastructure and retail access	[240,243,248]
**Educational Disparities**	Lower nutrition knowledge and food skills	Lower adherence to healthy dietary patterns	Nutrition education and community interventions	[245,249,250]
**Global Transferability**	Differences in culture, agriculture, and food availability	Challenges in adopting MedDiet outside Mediterranean regions	Adapt local diets using MedDiet principles rather than specific foods	[212,241,251]
**Policy & Structural Barriers**	Market failures, subsidies favoring commodity crops	Healthy foods appear less affordable than unhealthy options	Fiscal policies, agricultural reform, integrated food-system governance	[207,208,231,251]

## Data Availability

No new data were created or analyzed in this study. Data sharing is not applicable to this article.
